# Separation and Paired Proteome Profiling of Plant Chloroplast and Cytoplasmic Ribosomes

**DOI:** 10.3390/plants9070892

**Published:** 2020-07-14

**Authors:** Alexandre Augusto Pereira Firmino, Michal Gorka, Alexander Graf, Aleksandra Skirycz, Federico Martinez-Seidel, Kerstin Zander, Joachim Kopka, Olga Beine-Golovchuk

**Affiliations:** 1 Max Planck Institute of Molecular Plant Physiology, 14476 Potsdam-Golm, Germany; michal.gorka.ofc@gmail.com (M.G.); alex@smak-academy.de (A.G.); skirycz@mpimp-golm.mpg.de (A.S.); MSeidel@mpimp-golm.mpg.de (F.M.-S.); zander@mpimp-golm.mpg.de (K.Z.); kopka@mpimp-golm.mpg.de (J.K.); olga.beine@bzh.uni-heidelberg.de (O.B.-G.); 2 School of BioSciences, University of Melbourne, Melbourne, VIC 3010, Australia; 3 Heidelberg University, Biochemie-Zentrum, Nuclear Pore Complex and Ribosome Assembly, 69120 Heidelberg, Germany

**Keywords:** in vivo protein complex stabilization, plant proteomics, ribo-proteome, ribosome complexes, tissue specific separation

## Abstract

Conventional preparation methods of plant ribosomes fail to resolve non-translating chloroplast or cytoplasmic ribosome subunits from translating fractions. We established preparation of these ribosome complexes from *Arabidopsis thaliana* leaf, root, and seed tissues by optimized sucrose density gradient centrifugation of protease protected plant extracts. The method co-purified non-translating 30S and 40S ribosome subunits separated non-translating 50S from 60S subunits, and resolved assembled monosomes from low oligomeric polysomes. Combining ribosome fractionation with microfluidic rRNA analysis and proteomics, we characterized the rRNA and ribosomal protein (RP) composition. The identity of cytoplasmic and chloroplast ribosome complexes and the presence of ribosome biogenesis factors in the 60S-80S sedimentation interval were verified. In vivo cross-linking of leaf tissue stabilized ribosome biogenesis complexes, but induced polysome run-off. Omitting cross-linking, the established paired fractionation and proteome analysis monitored relative abundances of plant chloroplast and cytoplasmic ribosome fractions and enabled analysis of RP composition and ribosome associated proteins including transiently associated biogenesis factors.

## 1. Introduction

Ribosomes are highly conserved ribonucleoprotein complexes that translate messenger (m) RNA into proteins by ribozyme catalysis [1]. Owing to the complex endosymbiotic origin [2], plants unlike most other organisms have three types of ribosomes, namely prokaryote-type chloroplast or mitochondrial ribosomes and eukaryote-type cytoplasmic ribosomes, which assemble into mRNA decoding organelle 70S and cytoplasmic 80S monosome and polysome translation complexes. Ribosomes consist of small (SSUs) and large subunits (LSUs) that are synthesized as separate and initially translational inactive ribonucleoprotein complexes. The efficient assembly of eukaryote ribosomes in yeast requires equal quantities of four ribosomal (r) RNAs and 79 distinct ribosomal proteins (RPs) [3]. A multitude of ribosome biogenesis factors (RBFs) and small nucleolar RNAs act in highly regulated assembly and maturation processes that complete ribosome biogenesis in the cytosol. The final steps of cytoplasmic, that is, eukaryotic, ribosome biogenesis, appears to be in part conserved in plants [4,5,6]. Canonical cytoplasmic 40S SSUs contain 18S rRNA and 33 plant RPs. The plant cytoplasmic 60S LSU has 46 RPs and contains the 25S, 5.8S, and 5S rRNAs [7,8,9].

The recently elucidated structure of spinach chloroplast ribosomes confirms 25 prokaryote type SSU RPs that are assembled with 16S rRNA and 33 LSU RPs in a complex with 4.5S rRNA, 5S rRNA, and 23S rRNA. Plant 23S rRNA has two hidden breaks at positions 515 and 1755 [10], resulting in respective 23S rRNA fragments.

Several authors reviewed the complex pathways of eukaryotic ribosome biogenesis and specialized reviews were dedicated to the biogenesis of yeast, mammalian, and plant eukaryotic ribosomes [3,9,11,12,13,14,15,16,17,18]. The general mechanisms of 60S and 40S assembly are well conserved in eukaryotes [17]. Eukaryote ribosome biogenesis is compartmentalized and several distinct pre-rRNAs and pre-ribosomal complexes exist. Biogenesis starts in the nucleolus, continues in the nucleoplasm, and ends in the cytoplasm. In yeast, the first steps include independent transcription of 5S rRNA, and of a polycistronic 35S pre-rRNA. This transcript has a size of 45S in plants and of 47S in mammals. The pre-rRNA contains sequences of mature 18S, 5.8S, and 25S rRNAs. Ribosome assembly starts with a 90S rRNA–protein complex that still contains the joined precursors of the SSU (18S) and LSU rRNAs (25S and 5.8S). Successive assembly of proteins, rRNA folding, modification, processing, and cleavage occur by concomitant mechanisms. Following 90S assembly, the biogenesis pathways of 40S and 60S subunits are separated. Ribonucleoproteins generate 20S pre-rRNAs of the SSU branch and 27S A_2_ pre-rRNAs of the LSU branch. Assembly of RPs in the nucleus leads to pre-ribosomal subunits that are larger than the mature SSUs and LSUs, for example, the 66S-preribosomal subunit of the final 60S LSU branch. The two independent, but coordinated pathways culminate in the export of the yet immature pre-60S and pre-40S subunits from the nucleus to the cytoplasm. The last steps of 60S and 40S maturation occur in the cytoplasm, where protein factors protect and proof read yet immature subunits and facilitate the final transformation of pre-ribosomal complexes into translation-competent subunits.

A second level of complexity exists in plants that have multiple paralogs of each RP. Up to seven paralogs of each of the highly conserved RP families exist in *Arabidopsis thaliana* [19] and multiple yet non-assigned RP paralogs that are either annotated pseudogenes or protein coding. The genome of the model plant *Arabidopsis thaliana* (Arabidopsis) contains ~242 cytoplasmic RP genes [20]. More than 70 genes each encode plastid or mitochondrial RPs [21]. All of these factors contribute to a combinatorial universe of potential ribosome complexes [22] that may act redundantly or, as has been recently suggested, may be functionally specialized [23]. In view of the high number of plant paralogs, the plant universe of ribosomes may be exceptionally large. 

Ribosome preparation methods currently focus on purification and stabilization of actively translating polysome complexes for the purpose of ribosome profiling [24,25,26,27,28]. Ribosome profiling methods analyze mRNAs and mRNA footprints that are occupied by transcript decoding ribosomes [29]. Ribosome footprints, that is, mRNA sequences that are protected by translating ribosomes from experimental RNase digestion are thought to more accurately represent nascent protein synthesis than the currently widely applied proxy of steady-state transcriptome profiling by conventional total mRNA analysis methods. Ribosome profiling technologies have recently been adapted to plants in combination with nucleus- and protein-targeted capture techniques [30]. These methods allow paralleled profiling of nascent mRNA transcription in the nucleus and of ribosome-associated footprints from translated mRNA.

While translating ribosomes are in the current focus, investigations of plant ribosome biogenesis or heterogeneity are in their infancy. One crucial analytical tool that has not been available so far is the paired analysis of the non-translating and translating plant ribo-proteome. Such a tool will enhance our understanding of plant ribosome biogenesis and heterogeneity and enable analysis of developmental as well as cell- or stimulus-specific ribosome heterogeneity [31]. For this purpose, we selected Arabidopsis, the model of plant molecular biology. We optimized our methods towards improved resolution of plant ribosomal complexes. We analysed tissues from hydroponic Arabidopsis cultivation that support highly replicated and paired root and shoot tissue harvests in sufficient tissue amounts.

A good separation of non-translating subunits is a challenge and a useful way to assess the presence of RP paralogs in translating compared with non-translating ribosomes and of ribosome associated proteins, such as RBFs and translation factors, in these fractions. In this context, we aimed to characterize plant ribosome fractions separated by sucrose gradients. We focussed on the current study on non-translating ribosome complexes and especially the co-purification of 60S associated proteins from pre-60S complexes in non-cross-linked and cross-linked preparations.

**Figure 1:**
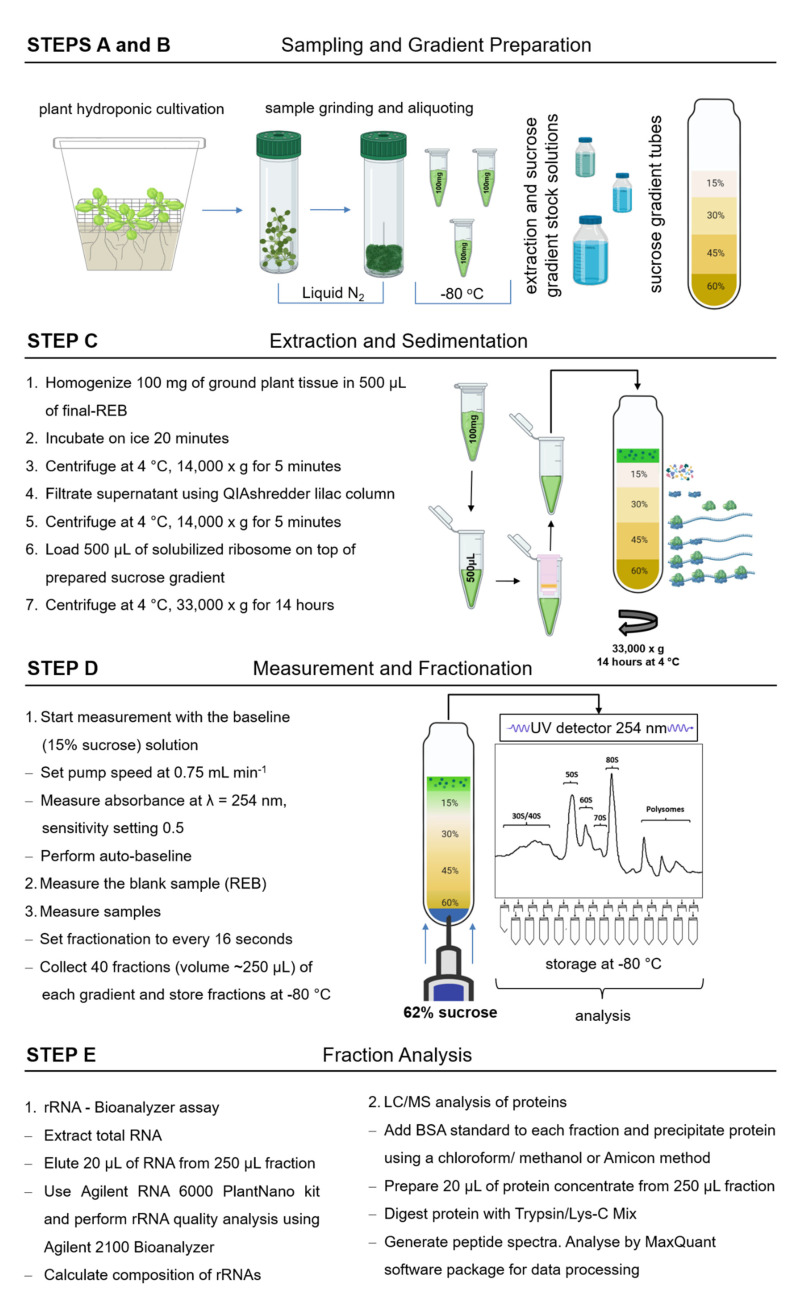
Schematic workflow of paired proteome profiling of non-translating and translating plant ribosome complexes. Steps include plant growth and sample processing (**A**), solution and sucrose gradient preparation (**B**), density gradient separation of macromolecular complexes (**C**), fractionation (**D**), and multiplexed analyses of resulting fractions (**E**). Details are reported in the Materials and Methods section. The figure contains objects created using a paid subscription of BioRender [32]. REB, ribosome extraction buffer; BSA, bovine serum albumin; LC/MS, liquid chromatography mass spectrometry.

In this report, we describe plant cultivation and sucrose density gradient based methods for the analysis of non-translating organelle and cytosolic ribosome complexes (Figure 1). Following the general workflow that was previously applied in replication to soil grown Arabidopsis rosettes [4], we characterized the separated organelle and cytoplasmic ribosome fractions from Arabidopsis leaf, root, and seed material by rRNA analyses. We selected leaf material for an in-depth proteomic analysis of the separated plastid and cytoplasmic ribosome complexes. We specifically applied our method to test for co-purification of low abundant ribosome associated proteins, such as RBFs that form immature ribosome biogenesis complexes or translation factors that are involved in initiation, elongation, or release. We describe the chemical stabilization of these transient complexes and provide snapshots of the late maturation steps of 60S LSU biogenesis. Finally, we discuss the potential of our workflow to enhance our understanding of the molecular physiology of organelle and cytoplasmic ribosome complexes and their respective RP composition.

## 2. Results and Discussion

### 2.1. Exemplary Profiles of the Plant Ribo-Proteome

We tested our established methods of ribo-proteome preparation and characterization using typical plant tissues of the model plant Arabidopsis. As a first example, we chose rosette leaves of soil grown plants [4] at the developmental stage ~1.10 [33]. This tissue contained both chloroplast and cytoplasmic ribosomes and was expected to have a highly complex composition of eukaryote- and prokaryote-type ribosome complexes. As a tissue with lower expected complexity, we chose root tissue [34] that we obtained from axenic cultures of hydroponically grown plants. This cultivation method was standardized and delivered whole root material from pooled samples of up to four co-cultivated plants (Figure S1). We analyzed root tissue of plants at developmental stage ~1.10. Leaf and root samples were harvested at ~6 h after dawn of a 16 h long day. As third example, we selected dry vernalized seeds of Arabidopsis before imbibition. Non-germinating seeds contain ribosomes in a non-active state [35] and no chloroplasts. Therefore, we again expected lower ribosome complexity compared with leaves. All plant material was obtained from plants that were cultivated under optimized growth conditions.

**Figure 2:**
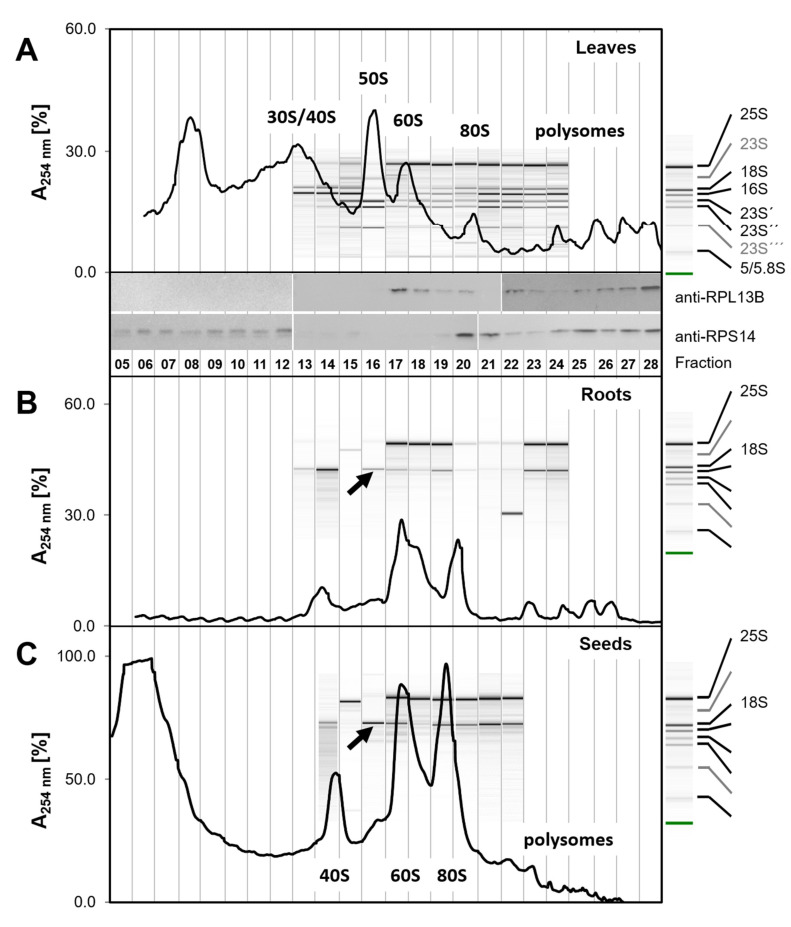
Comparative examples of ribosome sedimentation profiles from diverse plant tissues. Ribosomes were prepared from 100 mg (fresh weight) of *Arabidopsis thaliana* Col-0 rosette leaves (**A**) or roots (**B**) of plants at the developmental stage ~1.10 [33] and (**C**) from 50 mg dry vernalized seeds. Ribosome subunit profiling was performed as described in this study using a 15–60% sucrose gradient and monitored by blank gradient subtracted absorbance at λ = 254 nm. rRNA was analyzed and annotated by single-sample scaled microfluidic electrophoresis of total RNA according to [36]. Note that root and seed materials other than leaf contain predominantly cytosolic ribosomes, as indicated by the 25S and 18S rRNA annotations to the right of subfigures B and C. Western blots of independent leaf ribosome preparations were probed with anti-RPL13B (At3g49010) and anti-RPS14A (AT2G36160) antibodies. White separators indicate independently hybridized Western blots. Approximate positions of fractions and UV traces were aligned by gradient elution- and fractionation-times. Note the occurrence of 18S rRNA in fraction F16 that is present in preparations of root and seed material and faintly in leaf material (black arrows). Moreover, note the co-occurrence of chloroplast rRNAs in leaf fractions F15 and following (**A**); plastid rRNAs are very low abundant in non-green tissues (**B**,**C**).

**Figure 3:**
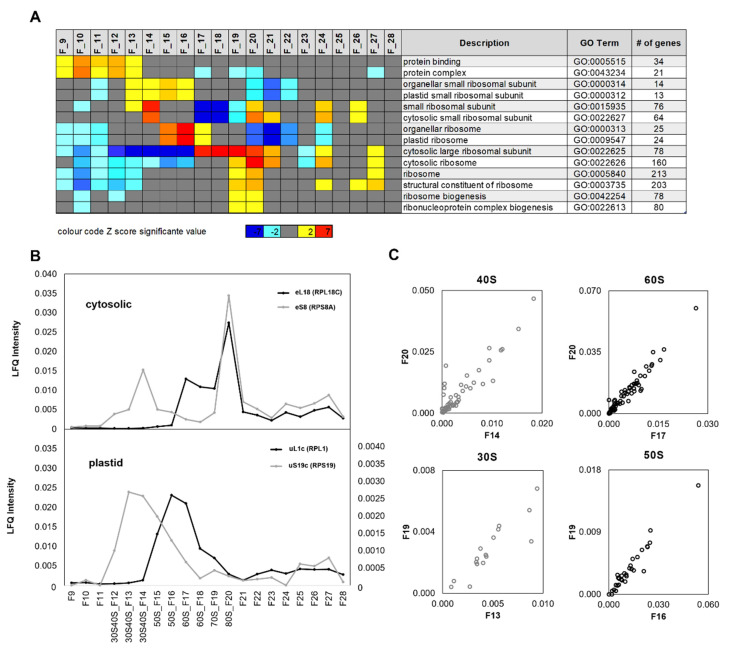
Proteomic characterization and assignment of sucrose density gradient fractions to non-translating and translating ribosome complexes. (**A**) Gene ontology (GO) enrichment analysis of log2-fold changes of bovine serum albumin (BSA)-normalized label-free quantification (LFQ) abundances across fractions F9–F28. Significant enrichments of gene ontologies across fractions F9–F28 are highlighted by color-coded Z-scores. Z-scores are color-coded blue (−7) to light blue (−2) to represent significant depletion and yellow (+2) to red (7) to represent significant enrichment of proteins belonging to the reported GOs. The numbers (gene #) of detected proteins from each GO are indicated in the final column. (**B**) Exemplary RP abundance profiles across fractions F9–F28 of ribosome complexes prepared from a representative leaf sample of *Arabidopsis thaliana*. LFQ abundances were normalized to a BSA standard added after gradient separation. Normalized LFQ abundances indicate the presence of chloroplast 30S (right axis) and 50S (left axis) ribosome subunits and the cytosolic 40S and 60S subunits in the diverse fractions. The corresponding UV trace of the analyzed density gradient is reported under Supplementary Material (Figure S2). Fraction assignments integrate proteome data and rRNA analyses (Figure 2), fraction F19 between the 60S and 80S complexes contains 70S monosomes. (**C**) Biplots of RP abundances from the indicated 80S (top) or 70S (bottom) monosome fractions over non-translating 40S or 60S fractions (top) and 30S or 50S fractions (bottom), respectively. According to the abundance maxima and shoulders of subfigure B, the monosomes were assigned to fractions F19 (chloroplast) and F20 (cytosolic) and the free subunits to fractions F13 (chloroplast 30S SSU), F14 (cytosolic 40S SSU), F16 (chloroplast 50S LSU), and F17 (cytosolic 60S LSU). The four sub-tiles show RPs that were correlated (Pearson´s correlation coefficient r ≥ 0.800) to the selected exemplary RPs of cytosolic 40S and 60S ribosome subunits or chloroplast 30S and 50S subunits reported in (B). Forty-six RPs correlated to RPS8A (AT5G20290) of the 40S eS8 family, 69 RPs correlated to RPL18C (AT5G27850) of the 60S eL18 family, 13 RPs correlated to RPS19 (ATCG00820) of the 30S eS19 family, and 30 RPs correlated to RPL1 (AT3G63490) of the 50S uL1c family (Supplementary Table S1).

We selected sucrose gradient fractions F05–F28 for comparative UV absorbance analysis (Figure 2). The rRNA composition of fractions F13–F24 of root and leaf material (Figure 2A,B) and of fractions F14–F22 of seed material (Figure 2C) was assessed by microfluidic analysis. Protein analysis focused on leaf material as the most complex tissue type. We performed initial qualitative Western blot analysis of leaf fractions F05–F28 (Figure 2A) and subsequent proteome analysis (Figure 3, Figure S2) by liquid chromatography mass spectrometry (LCMS) of fractions. The fractions for protein analyses were from independent preparations of leaf material. We aligned our diverse analyses across tissues and applied analysis technologies by gradient elution- and fractionation-time. This procedure enabled qualitative comparison, but the inaccuracy of drop-by-drop fractionation and slight differences of the sucrose gradients shifted in part intensity maxima across fraction borders.

### 2.2. UV Absorbance Analysis

As expected, UV absorbance traces of ribosome complexes from leaf material were highly complex in the region of both non-translating ribosomes and polysomes (Figure 2A). We inferred an overlay of chloroplast and cytosolic SSUs, LSUs, monosomes, and polysomes, but assignment of complex identity was not possible by UV analysis of this material alone. Comparison with the profiles of root and seed material lacking chloroplasts (Figure 2B,C) indicated the positions of the 40S SSUs, 60S LSUs, and 80S monosomes. The polysome region separated oligosome species, but our sucrose gradient system that was optimized for the region of non-translating complexes allowed monitoring of only the low oligomeric set of polysomes (Figure 2). Root preparations were low in contaminating non-ribosome protein complexes. Leaf preparations contained a characteristic peak in fractions F11–F14 that was absent from root material. Preparations from vernalized, but non-germinating seeds had a high background of low-density complexes and monomeric proteins in fractions F05–F07 and a very low abundant polysome region.

### 2.3. Microfluidic rRNA Analysis 

Microfluidic rRNA analysis of seed and root fractions covering F13–F24 confirmed UV (λ = 254 nm) based assignment of ribosome complexes to sedimentation fractions (Figure 2B,C). The low intensity or absence of chloroplast rRNA allowed unambiguous annotation of eukaryote-type 18S and 25S rRNAs. 18S rRNA indicated the accumulation of 40S SSUs in fractions F13–F15, F19–F20, and in high density fractions. 25S rRNA accumulated in fractions F17–F21 and higher fractions. Accordingly, we assigned fractions F13–F14 and the respective UV peak to non-translating 40S SSUs that may co-elute with 43S pre-initiation complexes [37,38,39], which are formed during an early step of eukaryotic translation initiation and consist of 40S bound by the initiation factors eIF1, eIF1A, eIF3, and the eIF2-Met-tRNA_i_^Met^-GTP ternary complex (eIF2-TC) [40]. Fraction F16 appeared to contain 48S preinitiation complexes [41] with a minor UV apex that was visible in fraction F16 of root and seed material (Figure 2B,C). The non-translating 60S LSU complexes were assigned to fraction F17 and the respective highly abundant UV apex. 80S monosomes accumulated in fractions F20–F21. The intermittent fraction F18 was high in 25S rRNA but low in 18S rRNA. Therefore, we assumed presence of 66S LSU maturation complexes in F18 [42,43]. Polysomes accumulated in fractions following F22, but were not baseline separated from 80S monosomes, especially in seed preparations. Additional unexpected components that were detected by microfluidic analysis in F15 of seeds and roots, with size smaller than 25S rRNA, or in root fraction F22, with size smaller than 18S rRNA, were not identified.

Ribosome preparations from leaves, in contrast to root and seed material, proved to generate a complex overlay of partially co-eluting chloroplast and cytoplasmic ribosome complexes (Figure 2A). Annotation of 16S rRNA and 23S rRNA was complicated by the presence of 23S post-maturation fragments, 23S´, 23S´´, and 23S´´´ and low abundance of non-cleaved 23S rRNA [36]. 16S rRNA accumulated in fractions F13–F15 and was present in fractions F18 and those following. 23S post-maturation fragments accumulated in fractions F15–F16 and were present in fractions F17 and those following. We concluded that chloroplast 30S SSUs with an UV apex in fractions F13–F15 co-eluted with non-translating 40S SSUs and likely 43S complexes (in the following 30S/40S fraction). The non-translating chloroplast 50S LSU complexes were separated from free 60S LSUs, but co-purified with 48S complexes. Similarly, the chloroplast 70S monosomes (F19) appeared to co-elute with 66S maturation complexes [13,42]. In the polysome fractions, low oligomeric chloroplast polysomes and cytosolic polysomes overlaid.

### 2.4. Western Blot Analysis

We confirmed the position of cytosolic 60S and 80S monosomes and polysomes in leaf ribosome sedimentation profiles by anti-RP specific ribosomal antibodies, namely anti-RPL13B (At3g49010) indicative of 60S LSUs and anti-RPS14A (AT2G36160) indicative of 40S SSUs (Figure 2A). RPL13B was present in fractions F17–F20 that were assigned to non-translating 60S LSU, 66S complexes, and 80S monosomes, and in all polysome fractions. The anti-RPS14A antibody verified the position of 80S monosomes and polysomes and indicated the presence of RPS14A in the co-eluting 30S/40S fractions and presence of RPS14A, likely as non-ribosome bound protein, in low-density fractions. Chloroplast or mitochondrial RPs were not tested by Western blot technology, but are part of the following ribo-proteome characterization. Because the PAGE and Western blot analyses of each protein were developed in parallel, the difference of RPS14 signals between F12 and F13 can be owing to technical issues and might not reflect the actual difference of protein amounts. The subsequent proteome analysis (Figure 3, Figure S2) by LC/MS of fractions from independent preparations of leaf material enabled comprehensive comparison and identification of proteins.

### 2.5. Characterization of the Ribo-Proteome 

Earlier studies that annotated the ribo-proteome based on genomic data reported 414 Arabidopsis genes that code for potential ribosomal proteins excluding pseudogenes and now obsolete gene models (Table 1). This set consisted of 242 genes of cytosolic RPs from 80 RP families [7] and included two small translated open reading frames of the RPL41 protein family [20,21]. Of the remaining genes, 76 genes of 58 protein families coded for plastid RPs [10,21]. Seventy-four previously annotated RP genes and 22 additional genes of 87 protein families that coded for mitochondrial RPs were recently described and confirmed by two independent studies that combined plant mitochondrial ribosome complex preparation with proteomic analyses [44,45]. For the purpose of our current study, additional RP candidates of mitochondrial RPs that were reported by only one of these studies were disregarded. Forty of the previously genome annotated genes remained unconfirmed sequence homologs of RPs and are addressed in the following as RP-homologs.

Our analysis of ribosome complexes from an Arabidopsis leaf sample yielded 216 RP proteins that were expected to be present in the sucrose density fractions of translating and non-translating ribosome complexes (Table 1). One hundred and forty-four RPs were of the eukaryote type (60S and 40S) and, except for some 60S LSU RP families, represented 74 of the expected 78 cytosolic RP families. Most of 40S SSU and 60S LSU RP families were represented by two or more of their known paralogs (Table 2). Fifty-eight proteins were chloroplast RPs and represented 55 of the 58 50S LSU expected chloroplast RP families. Only in the cases of uL18C (RPL18N) and bL19c (RPL19) was more than a single paralog of a chloroplast RP family detected (Table 2). Only a small number, 14, of 96 mitochondrial RPs and one of the previously annotated unconfirmed RP-homologs were detected (Table 1 and Table 2).

The low coverage of mitochondrial RPs in our analysis was expected and reflected the low LFQ signal abundance of mitochondrial RPs compared to the cytosolic and chloroplast RPs that are both abundant in leaf tissue (Figure 2). The detected mitochondrial RPs were all of the large subunit (mtLSU) and co-eluted with non-translating chloroplast 50S and cytosolic 60S complexes and with the polysome fractions ( Supplementary Figure S3, Supplementary Table S1). Our current study did not allow a further analysis of mitochondrial RPs from leaf material. Low abundance of small and large subunit mitochondrial RPs was reported by Cheong and co-workers, too [34]. In this study of root material plastid and mitochondrial ribosomes co-purified and both prokaryote-type ribosome complexes were on average more than 100-fold less abundant than eukaryote ribosome complexes [34]. The mean LFQ abundance of RPs from the small mitochondrial subunit (mtSSU) was much lower than of mtLSU RPs [34]. This observation allowed us to conclude that our analysis of leaf material likely missed the small subunit mitochondrial RPs because of their low signal abundance. Due to the low abundance and diversity, mitochondrial ribosomes are difficult to study. They are known to have variable sedimentation coefficients, composition or protein:RNA mass ratios. Indeed, diverse types of subunits exist in different organisms [46,47,48]. Much of our current knowledge is derived from yeast, trypanosomatid or mammalian organisms. Several studies indicate that plant mitochondrial ribosomes considerably differ from bacterial ribosomes and from other eukaryotic mitochondrial ribosomes. They appear to have a higher molecular weight, an additional rRNA domain and appear to contain non-canonical proteins like peptidases, proteases and Pentatricopeptide Repeat (PPR) proteins [44,45,49,50].

Besides RPs, we detected a multitude of additional proteins that, in most cases, did not co-purify with ribosome complexes and can be considered purification contaminants. These proteins were present predominantly in the low-density fractions F9–F11 (Figure 3A). Gene ontology (GO) enrichment analysis of all detected proteins using log_2_-fold changes of protein LFQ abundances across fractions F9–F28 (Figure S2) confirmed separation of ribosome complexes from non-ribosomal proteins except for a purification-overlap zone in F13 of chloroplast 30S SSU RPs and non-ribosomal proteins. This overlap was exemplified by the gene ontologies, protein binding (GO:0005515), and protein complex (GO:0043234), with 34 and 21 proteins detected in our study, respectively, compared with plastid 30S SSU gene ontology, GO:0000312 (Figure 3A). Chloroplast 30S SSU RPs were enriched across fractions F13–F16. Further chloroplast RPs, GO:0009547, were enriched in fraction F15–17, indicative of non-translating 50S LSU complexes. Assembled 70S monosomes and chloroplast monosomes were not detectable by enrichment analysis. The set of detected RPs was predominantly cytosolic and of the eukaryote type (Table 1). Accordingly, 40S SSU RPs were found to be highly enriched in fraction F14, as well as in fractions F20–21, F24, and F26. This pattern indicated non-translating 40S (F14), assembled 80S (F20–F21), and two cytoplasmic polysome fractions. 60S LSU RPs were enriched across fractions F17–F21, comprising first non-translating 60S complexes (F17–F18) followed by assembled 80S monosomes (F20–F21). In addition, 60S RP enrichment was detectable in polysome fractions F24 and F27. Ribosome biogenesis related proteins were enriched in fractions F19–F20 and indicated 66S LSU or larger ribosome maturation complexes.

Enrichment analysis did not reflect abundance profiles of RPs across fractions F9–F28 and was limited to the predefined GO terms and corresponding gene sets. For analysis of abundance profiles, we arbitrarily selected representative proteins of the cytosolic and chloroplast, small and large ribosome subunits (Figure 3B). Protein eS8 (RPS8A), part of the 40S SSU, had abundance maxima in fraction F14 and F20 and confirmed annotation of the non-translating 40S SSU and assembled 80S factions, as well as the presence of cytosolic polysomes in fraction F24 and those following. eL18 (RPL18C) of the 60S LSU correlated to eS8 (RPS8A) specifically across F19–F28 and confirmed the fraction assignments of 80S monosomes and cytosolic polysomes. The chloroplast uS19c (RPS19) of the 30S SSU confirmed the broad distribution of non-translating 30S SSUs across sucrose density fractions F12–F15 with an apex in F14. An abundance apex of low intensity in F19 indicated assembled 70S monosomes. Increasing abundance in the following fractions confirmed the presence of chloroplast polysomes (Figure 3B). Chloroplast uL1c (RPL1) of the 50S LSU confirmed the presence of non-translating 50S LSUs in fractions F16–F17 and, by an abundance shoulder, the presence of 70S monosomes in fraction F19. Chloroplast polysomes were also indicated in fractions F22 and those following.

Using the four selected representative RPs as “baits”, we applied correlation analysis across the sucrose density fractions to search for other RPs that were part of the large and small cytosolic and chloroplast ribosome subunits. Pearson´s correlation of BSA-normalized LFQ abundances of all detected proteins across fractions F9–F28 revealed 158 “prey” proteins (Table S1) that were highly correlated to either of the four reference proteins (Pearson´s correlation coefficient r ≥ 0.800). Forty-six RPs correlated to RPS8A (AT5G20290) of the 40S eS8 family, 69 RPs to RPL18C (AT5G27850) of the 60S eL18 family, 13 RPs to RPS19 (ATCG00820) of the 30S uS19c family, and 30 RPs to RPL1 (AT3G63490) of the 50S uL1c family. The correlation of 60S proteins to RPS8A and 40S proteins to RPL18C was very low, at 12.85% and 10.63%, respectively. This is owing to the detection of these proteins in 80S and polysome fractions. Thereby, we confirmed that the chosen reference proteins were indeed representative of their respective 30S, 50S, 40S, and 60S ribosome subunits. Additional detected RPs (Table 2) were correlated by Pearson´s correlation coefficient r < 0.800 or owing to low abundance present only in a subset of fractions.

On the basis of the abundance maxima (Figure 3B), we checked if the 158 cytosolic or chloroplast RPs and the four “bait” proteins were correlated between the respective non-translating subunit fractions and their corresponding monosome fraction. For this purpose, we plotted the normalized LFQ intensities (Table S1) of fractions F20 (cytosolic 80S) and F19 (chloroplast 70S) against the normalized LFQ intensities of the subunit fractions, F14 (cytosolic 40S SSU), F17 (cytosolic 60S LSU), F13 (chloroplast 30S SSU), and F16 (chloroplast 50S LSU) (Figure 3C, top and bottom). The 30S and 40S complexes extended across several fractions and were not separated, and we chose to visualize the correlation of F13 (30S) to F19 (70S) and of F14 (40S) to F20 (80S), based on the abundance maxima and shoulder positions of the reference protein profiles (Figure 3B). Because 30S and 40S fractions are not well-separated, the correlation of the alternate fraction combinations, for example, F13 × F20 (30S × 80S) and F14 × F19 (40S × 70S), was similar (Table S1).

### 2.6. Ribosome Biogenesis Factors

**Figure 4:**
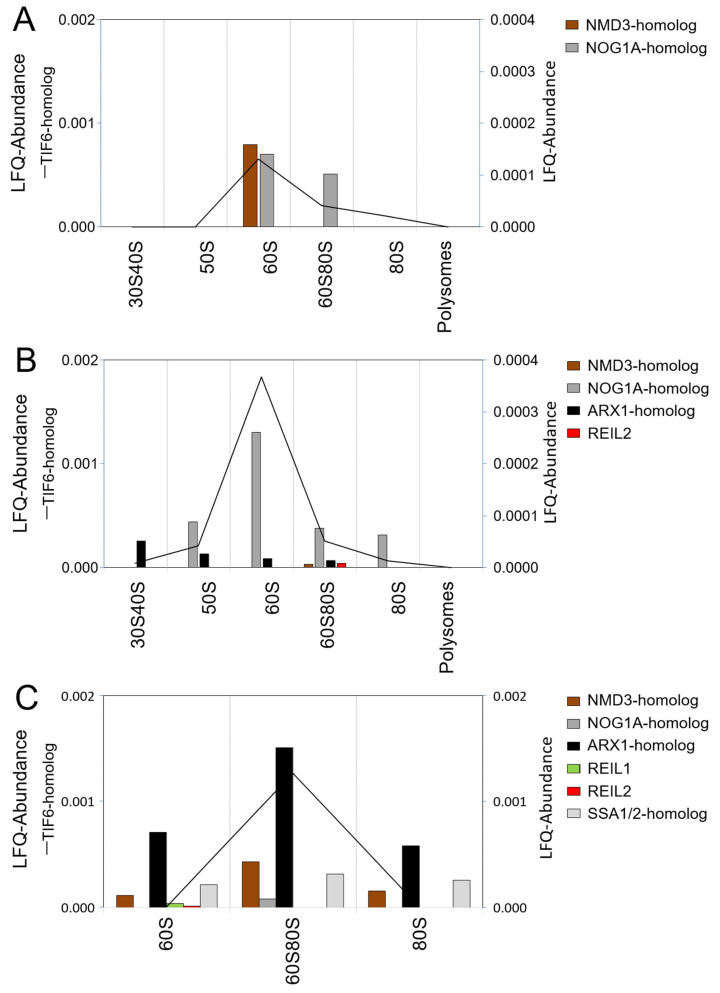
Co-purification selected Arabidopsis ribosome biogenesis factors homologs with ribosome complexes separated by sucrose density gradient centrifugation. Density fractions of ribosome preparations from *Arabidopsis thaliana Col-0* rosette leaves were combined as indicated in Figure 3B and submitted to proteomic analysis. The 60S80S fraction is equivalent to F19 and, in addition, contained 70S monosomes. BSA-normalized LFQ abundances are reported. Abundance of the Arabidopsis TIF6-homolog is represented as a line plot (left y-axis), and the remaining abundances as bar-plots (right y-axis). (**A**) Methanol/chloroform precipitation of ribosome complexes prepared from non-cross-linked Arabidopsis leaf tissue. (**B**) Purification and concentration of ribosome complexes prepared from non-cross-linked Arabidopsis leaf tissue by AMICON 3 kDa cutoff ultra-membrane centrifugation. (**C**) Methanol/chloroform precipitation of ribosome complexes prepared from Arabidopsis leaf tissue that was in vivo cross-linked by 0.5% formaldehyde.

**Figure 5:**
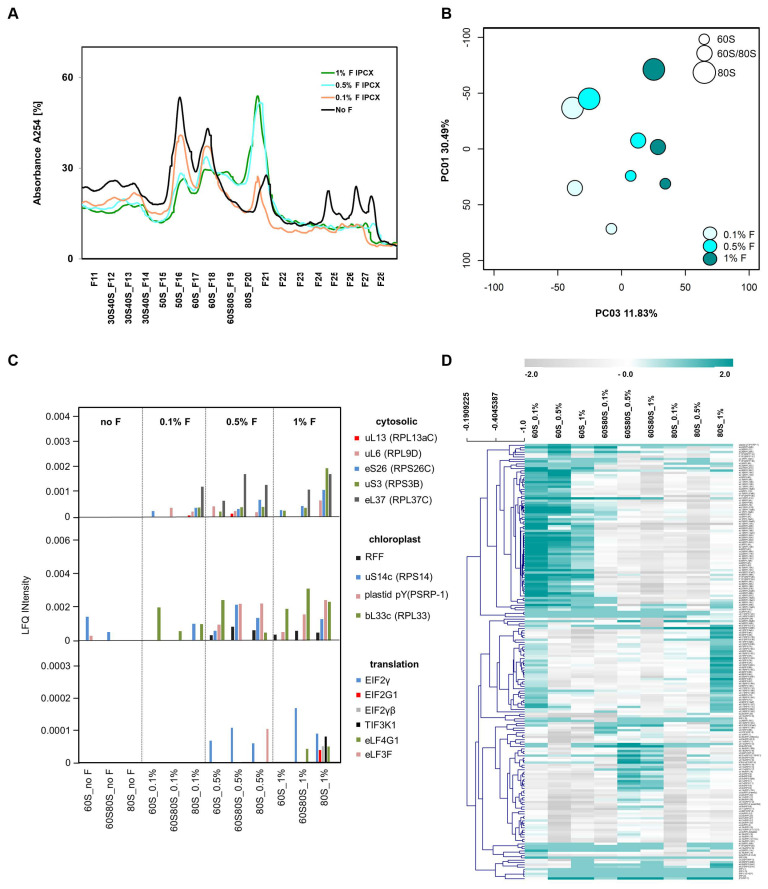
Analysis of changes of ribo-proteome profiles induced by in vivo formaldehyde crosslinking. (**A**) UV-absorbance profiles of non-cross-linked compared with in vivo crosslinking by 0.1%, 0.5%, and 1.0% (*v*/*v*) formaldehyde. Note loss of polysomes and accumulation of monosomes. (**B**) Principal component analysis (PCA) of a proteomic analysis of the 60S, 60S80S intermediate fraction, and 80S fraction. Changes of log_2_-transformed relative LFQ abundance changes of all annotated RPs relative to the corresponding non-cross-linked samples were analyzed. Note PC3, with 11.83% contribution of the formaldehyde treatment to total RP variance. PC2 (not shown) represented 20.15% of total variance and distinguished the intermediate fraction 60S80S from the 60S and the 80S fractions. (**C**) LFQ abundances of cytoplasmic RPs, chloroplast RPs, and translation factors that became detectable by in vivo formaldehyde crosslinking. Note accumulation of translation factors in the 80S fraction. (**D**) Heat map of the proteomic analysis and hierarchical cluster analysis (HCA) using average linkage. The grey (−2) to cyan (+2) color scale codes for the log_2_-transformed ratios of RP abundances relative to the corresponding non-cross-linked fractions. Refer to Figure S4 for higher resolution.

UV-based ribosome sedimentation profiles and rRNA analysis indicated the presence of transient ribosome complexes in our preparations (Figure 2). In the following, we investigated the presence of 60S ribosome biogenesis complexes that we expected to co-purify with non-translating 60S complexes and 70S or 80S monosomes in fractions sucrose density fractions F17–F21 (Figure 2 and Figure 3A). We focused on 60S LSU assembly that entails transient binding of protein factors in the nucleus to form 66S complexes and subsequent export of pre-60S complexes from the nucleus to the cytosol, where the last maturation steps of translationally competent 60S LSUs occur. To analyze these complexes, we compared non-pretreated ribosome preparations to preparations that were chemically cross-linked (Figure 4 and Figure 5). We argued that 60S biogenesis complexes might be short-lived or unstable during preparation. To stabilize transient protein–RNA and protein–protein interactions, different in vivo cross-linking methodologies like phenol-toluol extraction or UV light irradiation exist and were successfully applied [52,53,54,55,56]. We chose an in vivo crosslinking approach that applied in vivo formaldehyde permeation to Arabidopsis rosettes that were freshly harvested. Formaldehyde is a so-called ‘zero-length’ reversible cross-linker that establishes covalent bonds between amino acid residues across a ~2 Å distance and, thereby, is thought to minimize accidental crosslinks owing to non-specific protein interactions [57].

To test the stabilization and presence of pre-60S complexes in our preparations from leaf tissue, we selected nine Arabidopsis candidate proteins. The selection was by homology to yeast ribosome biogenesis factors that are thought to be involved in cytosolic 60S maturation and quality control steps [12,58,59,60]. The selection consisted of Arabidopsis homologs of NMD3 (AT2G03820), TIF6 (AT3G55620), two NOG1-homologs (NOG1A, AT1G50920, and NOG1B, AT1G10300), ARX1- (AT3G51800) and JJJ1-homologs (AT1G74250), REIL1 (AT4G31420), REIL2 (AT2G24500), and one of several SSA1/2-homologs (AT3G09440) [5].

The Arabidopsis TIF6-homolog was the most abundant ribosome biogenesis factor across analyses and accumulated in the 60S fraction or the intermediate fraction, in the following 60S/80S fraction, between the non-translating 60S LSUs and 80S monosomes (Figure 4). Methanol/chloroform precipitation of ribosome complexes prepared from non-cross-linked Arabidopsis leaf tissue yielded only the NMD3 and the NOG1A-homolog next to the TIF6-homolog (Figure 4A). Replacement of precipitation by purification of ribosome complexes and concentration by AMICON ultra-membrane centrifugation revealed the presence of the ARX1-homolog and REIL2 and an increase of TIF6- and NOG1A-homolog abundance (Figure 4B). In vivo formaldehyde crosslinking of leaf tissue combined with methanol/chloroform precipitation of ribosome complexes stabilized pre-60S ribosome complexes and seven of the nine selected candidate proteins were detectable, namely, the TIF6-, NMD3-, NOG1A-, ARX1-, REIL1, REIL2, and SSA1/2-homolog (AT3G09440) (Figure 4C). The TIF6-, NMD3-, NOG1A-, ARX1-, and SSA1/2-homolog accumulated in the cross-linked 60S/80S fraction, and REIL1 and REIL2 were detectable in the cross-linked 60S fraction. Our methods failed to demonstrate the presence of the JJJ1- and NOG1B- homologs. REIL proteins were detectable at low abundance. Without cross-linking, REIL2 became detectable in the 60S80S fraction after sample concentration by ultra-membrane centrifugation. Both REIL paralogs were detected after cross-linking. Cross-linking, however, shifted REIL proteins to the 60S fraction and appeared to preferentially stabilize a small pre-60S complex. The concomitant shift of TIF6-, NMD3-, NOG1A-, and ARX1-homologs to the larger 60S80S fraction and their change in relative abundance provided further evidence of changes among the stabilized pre-complexes. Crosslinking appears to capture pre-60S or 66S complexes that are instable and possibly artificial or too short lived in native state. Clearly, further experimental efforts are required to enrich and prepare native REIL–60S complexes for structural analysis.

In vivo formaldehyde crosslinking stabilized and enriched pre-60S ribosome biogenesis complexes, but altered the ribosome profile dependent on formaldehyde concentration. We tested formaldehyde crosslinking by 0.1%, 0.5%, and 1.0% (*v*/*v*) and analyzed the proteome of the 60S, 60S/80S, and 80S fractions (Figure 5A). Principal component analysis (PCA) of the ribo-proteome of these fractions indicated that 30.5% of total proteome variance constituting PC1 was explained by the separated ribosome fractions (Figure 5B), but 11.8% of ribo-proteome variance (PC3) in our experiment was generated by increasing the formaldehyde concentration. In vivo formaldehyde crosslinking caused loss of polysomes (Figure 5A). 80S monosomes and the 60S/80S fraction increased at formaldehyde concentrations ≥0.5% (*v*/*v*) with concomitant reduction of all non-translating fractions. Apparently, in this treatment, there was a polysome run-off and 80S monosomes were arrested in preparations from Arabidopsis leaves. These phenomena were further supported by a heat map and hierarchical clustering of RP abundances (Figure 5D, and a higher resolution version in Figure S4). We tried to transfer the crosslinking from yeast or mammalian cell cultures to plant tissue by in vivo vacuum infiltration. Although cycloheximide was used in the extraction buffer and throughout the subsequent sedimentation process, apparently, the vacuum infiltration of plant tissue is too slow. Likely, the plant tissue and cell wall material causes tissue penetration to be inefficient. We do not recommend in vivo cross-linking following the procedures described in this manuscript and, for now, recommend analysis without crosslinking. However, we can envision variations of the crosslinking procedure, for example, shock-freezing of plant tissue, frozen grinding, and in vitro cross-linking, that are worthwhile to explore in the future.

Three main clusters indicated decreasing relative amounts of RPs in the 60S fraction and increasing RP abundances in the 80S and 60S/80S fractions. Few cytosolic and chloroplast RPs that were not detectable without crosslinking became apparent likely owing to increased ribo-proteome abundance in the 60S/80S and 80S fractions (Figure 5C). Accumulation of chloroplast RPs indicated that formaldehyde crosslinking also arrested the 70S monosome (Figure 5C,D). Finally, translation initiation factors and translation elongation factors became detectable by crosslinking. Translation initiation factors, however, appeared not to dissociate and remained cross-linked mostly to 80S monosomes (Figure 5C).

## 3. Materials and Methods

The preparative and analytical workflow that we established and describe in the following requires two days to prepare stock solutions and ultracentrifugation tubes with pre-formed sucrose gradients (Figure. 1). Two additional days are required for extraction, separation, and fractionation of ribosome complexes by density gradient sedimentation of plant samples. Subsequent days may include RNA and protein preparation for micro-fluidic rRNA analyses, Western analyses, or proteome analysis. 

### 3.1. Plant Cultivation, Sampling, and In Vivo Protein Crosslinking

#### 3.1.1. In Vitro Plant Cultivation

We developed an easy to manifold and handle hydroponic cultivation system for Arabidopsis plants that can be routinely autoclaved by dry heat. This cultivation system enabled paired sampling of shoot and root material under controlled sterile conditions (Additional File 1: Figure S1). Circular glass cultivation pots of 8 cm diameter were fitted with a planar circular mesh insert (in the following, “trampoline”) that was folded and fitted to size from a circular cutting of 11 cm diameter of stainless-steel mesh of 0.25 mm wire diameter and 1.4 mm mesh width, for example, “Edelstahl Drahtgewebe Fliegengitter Gaze” [61]. The rim of the trampoline was bent down towards the bottom of the glass pot and fixed by material tension. Each pot was filled with 250 mL liquid Murashige and Skoog (MS) medium containing 2% (*w*/*v*) sucrose [62]. The trampoline was adjusted to the liquid top, avoiding seedling submergence and air bubbles below the mesh. Seeds were placed onto sterile and equally spaced small pieces of agar-solidified MS medium, 0.8% (*w*/*v*) agar, and 2% (*w*/*v*) sucrose. Four seedlings per pot were either directly germinated on the sterilized trampoline or pre-germinated and transferred at developmental stage ~1.01–1.02 [33]. Plantlets were harvested at stage ~1.10 [33] after approximately four weeks at 16 h/8 h (day/night), 20 °C/18 °C temperature (day/night), 150 µmol m^−2^ s^−1^ light intensity, and ~100% relative humidity with loosely fitting glass lids. The liquid filled bottom of the glass pots was darkened by non-transparent plastic racks.

#### 3.1.2. Sampling

*Arabidopsis thaliana* Col-0 plant material, namely whole shoot or root systems, were pooled per glass pot from 3–4 plantlets to generate a single biological replicate of each tissue. Surplus liquid medium was removed rapidly by filter paper. Plant material was harvested into 2 mL round bottom micro-centrifuge tubes that contained a 5 mm diameter stainless steel ball. Samples were shock-frozen in liquid nitrogen within less than or equal to 10 s after dissection. Samples were homogenized by an oscillating ball mill [63] by two 1 min oscillation bursts at 25 Hz. Analyses of ribosome complexes were performed with 100 mg fresh weight (FW) starting material of leaf or root tissue or with 50 mg dry weight (DW) of seed tissue.

#### 3.1.3. In Vivo Protein Crosslinking

Routine preparation of ribosome complexes was performed without chemical crosslinking. To stabilize short-lived ribosome complexes and weak or transient protein-ribosome interactions, we used reversible chemical crosslinking by formaldehyde [64]. For this purpose, whole plant rosettes were harvested into 25 mL of ice-cold 100 mM sucrose, 50 mM NaCl, in 10 mM sodium phosphate buffer adjusted to pH 7 (MC buffer). MC buffer contained either 0.1 % (*v*/*v*), 0.5 % (*v*/*v*), and 1.0 % (*v*/*v*) formaldehyde, or no formaldehyde for control purposes. Whole rosettes were vacuum infiltrated on ice by applying 24 mbar vacuum twice for 5 min with intermittent gentle mixing and surface moistening of not fully submerged tissue. Crosslinking was stopped by adding 2.5 mL of 1.25 M glycine and 5 min vacuum infiltration. Plant tissue was washed three times with formaldehyde-free ice-cold MC buffer. Finally, adherent residual liquid was removed by paper towels. The plant tissue was shock-frozen in liquid nitrogen and kept frozen during homogenization by an oscillating ball mill, as described above.

### 3.2. Preparation of Ribosome Complexes

#### 3.2.1. Buffers and Solutions

Pre-ribosome extraction buffer (REB) and stock solutions that are required for the final, ready to use REB (Table 3) were prepared in double distilled-water (ddH_2_O) treated with diethyl pyrocarbonate (DEPC), if not indicated otherwise. All solutions were prepared under sterile conditions using sterilized tubes and pipet tips under a laminar flow bench. The following solutions were autoclaved and stored at room temperature (convenient stock-volumes are reported in square brackets): 2 M Tris-HCl pH 9.0 [1 L], 2 M KCl [1 L], 0.5 M ethylene glycol-bis (β-aminoethyl ether)-*N*,*N*,*N*′,*N*′-tetraacetic acid (EGTA) pH 8.3 [0.5 L], 1 M MgCl_2_ [0.5 L], 20% (*v*/*v*) polyoxyethylene 10 tridecyl ether (PTE) [25 mL], and 10% sodium deoxycholate (DOC) [25 mL]. A 20% detergent mixture [50 mL] consisted of 20% (*w*/*v*) Brij-35, 20% (*v*/*v*) Triton X-100, 20% (*v*/*v*) Igepal CA 630, and 20% (*v*/*v*) Tween 20. This mixture was initially heated to not more than 60 °C and dissolved completely. Before dispensing volumes for REB preparation, all stock solutions were gently mixed avoiding bubble formation. The 20% detergent mixture was re-heated to 45 °C to ensure complete and homogenous solution of all components.

The above-described stock solutions were combined to obtain pre-REB using volumes and temperatures reported in Table 3. Pre-REB was aliquoted into 5 mL portions stored at −20 °C until further use. The ice-cold final REB was prepared freshly on each day of extraction (Table 3) by adding the following stock solutions to 5 mL of thawed-on-ice pre-REB (convenient stock-volumes are reported in square brackets): 1 M dithiothreitol (DTT) [100 mL], 50 mg mL^−1^ cycloheximide [50 mL in ethanol], 50 mg mL^−1^ chloramphenicol [50 mL in ethanol], 200 mg mL^−1^ heparin [1 mL], 0.5 M phenylmethylsulfonyl fluoride (PMSF) [15 mL in isopropanol], and 1 tablet mL^−1^ cOmplete^TM^ protease inhibitor mixture (Sigma-Aldrich, St. Louis, MO, USA). These solutions were prepared without autoclaving and stored at −20 °C. Before pipetting, these solutions were carefully mixed avoiding bubble formation and kept on ice. Cycloheximide, chloramphenicol, heparin, and DTT inhibit eukaryotic and chloroplast translation elongation by arresting ribosomes. PMSF and cOmplete^TM^ protease inhibitor mixture suppress protease activities.

#### 3.2.2. Extraction of Ribosome Complexes 

The ribosome extraction procedure was according to previously reported protocols [65,66] with slight modifications. In detail, 100 mg (FW) frozen and pulverized plant tissue or 50 mg (DW) frozen and homogenized seeds were mixed with 0.5 mL pre-cooled, freshly prepared final REB. Frozen plant material and REB were gently mixed using a pipette tip until thawing was complete. This and all of the following steps were performed on ice or at ≤4 °C. Samples were incubated 20 min with gentle shaking at 35 rotations per minute. To avoid debris, the supernatant was centrifuged 2 min at ~14,000× *g* and 4 °C. The supernatant was loaded onto a lilac QIAshredder mini spin column (Qiagen, Hilden, Germany), centrifuged and the eluate was loaded onto previously prepared sucrose gradients in ultracentrifugation vials (cf. below).

#### 3.2.3. Preparation of Sucrose Gradient Solutions 

Sucrose density gradients were pre-formed in ultracentrifugation tubes (Table 4) and stored at −80 °C until further use. The following stock-solutions were required to prepare sucrose gradient solutions (convenient stock-volumes in reported in square brackets). A solution of 10× salt and buffer [100 mL] contained 20 mL of 2 M Tris-HCl, 10 mL of 2 M KCl, and 20 mL of 1 M MgCl_2_ (Table 3). After pH was adjusted to 8.4 with 1 M HCl, ddH_2_O was added to adjust the final volume of 100 mL. The completed solution was autoclaved and stored at room temperature.

2M sucrose, that is, 68.5% (*w*/*v*), was dissolved in ddH_2_O using a water bath set to less than 50 °C. The dissolved final sucrose solution was filtered using 0.22 µm qpore sterile filtration unit (Neolab, Heidelberg, Germany) and stored at room temperature. Prior to gradient assembly, four sucrose gradient solutions were prepared with concentrations of 15% (*w*/*v*), 30% (*w*/*v*), 45% (*w*/*v*), and 60% (*w*/*v*) sucrose. The volume parts of the 2 M sucrose stock-solution, the 10x salt and buffer solution, ddH_2_O, cycloheximide, and chloramphenicol solutions that are required for 1.8 mL of 15% and 60% sucrose step gradient solutions and for 3.6 mL final volumes of the 30% and 45% sucrose solutions are reported in Table 4. The reported volumes are sufficient for the assembly of 9 mL large-volume or 4.5 mL small-volume final sucrose gradients.

#### 3.2.4. Assembly of Sucrose Gradients 

Sucrose gradients were assembled in large (13.2 mL) or small (5 mL) thin-wall polypropylene ultracentrifugation tubes (Beckman Coulter, Krefeld, Germany, reference numbers 331372 or 326819). Gradient assembly is, in our hands, the critical step for reproducible separation of ribosome complexes. To ensure reproducible gradient performance, we recommend in-parallel preparation of large sets of sucrose gradient ultracentrifugation tubes from the same 15%, 30%, 45%, and 60% (*w*/*v*) sucrose gradient solutions that are best prepared in large volumes. These prepared volumes of sucrose gradient solutions should ideally be sufficient for at least 50 large or small ultracentrifugation tubes.

All preparation steps were performed in a cold room, unless indicated otherwise. All sucrose gradient solutions were temperature equilibrated to 4 °C and thoroughly mixed before pipetting. The only exception was the 60% solution, which, owing to the high viscosity and for proper dispensation, was equilibrated and pipetted at room temperature. The four sucrose concentrations steps were pipetted in ascending order, starting with the 60% (*w*/*v*) sucrose gradient solution. The volumes for large and small ultracentrifugation tube are reported within Table 4. After the 60% step, the ultracentrifugation tubes were pre-cooled at −80 °C, transferred to the cold room for subsequent pipetting, and re-frozen 15–20 min at −80 °C before adding the next concentration step. We took care to dispense exactly reproducible volumes of the in part highly viscous solutions. For this purpose, we used a single pre-moistened pipet for each concentration step, that is, we discarded the first volume from the yet non-wetted pipets. Pipetting of each concentration step into the set of ultracentrifugation tubes should be continuous without interruption. Intermittent thawing of the already frozen material and hoarfrost deposition between pipetting steps must be avoided.

#### 3.2.5. Sample Loading and Ultracentrifugation

The extract of ribosome complexes, that is, the eluate of QIAshredder mini spin columns, was loaded without delay onto the sucrose gradients prepared in ultracentrifugation vials. Prior to loading, the prepared sucrose step gradients were taken from −80 °C storage and kept cold at 4 °C overnight, to allow diffusion and formation of a continuous gradient. Any abrupt movement or vibration while handling the thawing and thawed tubes must be avoided to minimize gradient perturbation. The extracts of ribosome complexes or 500 µL REB non-sample controls, that is, blanks, were loaded gently and in steps with a 100 µL or 200 µL pipet onto the top of the thawed sucrose gradients by letting the liquid run slowly down the tube walls. The surface of the sucrose gradients must not be perturbed by rapid, vigorous pipetting. All sample tubes of a run were equally balanced using REB. We centrifuged with either a SW41Ti rotor of 13.2 mL nominal tube capacity or using a SW55Ti rotor of 5.0 mL nominal tube capacity. The rotors were swinging-bucket and operated by an Optima L80-XP ultracentrifuge (Beckman Coulter, Krefeld, Germany). The rotors are limited to six buckets per ultracentrifugation run. Each run had a non-sample gradient overlaid with 500 µL REB and a gradient with a reference preparation of ribosome complexes from Arabidopsis rosette leaves at developmental stage 1.10 [33] for quality control and four varying experimental extracts, as reported previously [4]. Ultracentrifugation was performed either for 14 h at 33,000× *g* and 4 °C using the SW41Ti rotor (Beckman Coulter, Krefeld, Germany) or 2 h at 50,000× *g* and 4 °C using the SW55Ti rotor (Beckman Coulter, Krefeld, Germany), as previously described [4,34]. We selected the slowest acceleration and deceleration programs, which were of ~6 min duration with transition from slow to maximum acceleration/deceleration starting at 500 rpm.

#### 3.2.6. Fractionation of Sucrose Gradients

After ultracentrifugation, sucrose gradients with separated ribosome complexes were retrieved from the top of bottom-pierced ultracentrifugation tubes. Approximately 250 µL fractions were collected at 0.75 mL min^-1^ flow rate with continuous absorbance measurement at λ = 254 nm using a Brandel BR-188 density gradient fractionation system (Alpha Biotech Ltd, Glasgow, UK). Considering the solvent delay between the UV-detector and fractionator, ~100 s equivalent to 1.25 mL, up to 40 fractions were collected at room temperature from large ultracentrifugation tubes, and up to 20 fractions from small tubes. After completion of fractionation, the collected ~250 µL fractions were stored at −80 °C until further use.

In detail, prior to the sampling of each of the six 15–60% (*w*/*v*) sucrose gradients from a rotor set, the fractionation system was equilibrated by first mounting a clean ultracentrifugation tube filled to approximately 80% of the volume with 15% (*w*/*v*) sucrose gradient solution. After piercing the clean tube at the bottom, the 15% (*w*/*v*) solution was pumped through the system and an absorbance baseline was recorded. Subsequently, a 15–60% (*w*/*v*) sucrose gradient tube was mounted and pierced at the bottom and high-density chase solution was used to push and empty the gradient from the bottom to top. The chase solution contained 62% (*w*/*v*) sucrose in 1x salt and buffer solution, ddH_2_O, and 1 µg mL^−1^ bromophenol blue. This solution was filtered using 0.22 µm pore size sterile filters and stored at 4 °C. Air bubbles must be avoided when mounting the tubes and preparing the fractionation system. Before and after fractionation of each gradient, the system is best cleaned sucrose-free by flushing the system in both forward and backward flow mode with at least 10 mL DEPC–water.

### 3.3. Ribosome Fraction Identification and Protein Cleanup

#### 3.3.1. RNA Extraction and Analysis

Total RNA extracts were prepared using guanidinium thiocyanate-phenol-chloroform extraction with TRIzol^TM^ (Thermo Fischer, Waltham, MA, USA). Briefly, 350 µL of TRIzol and 150 μL of chloroform/isoamylalcohol (*v*/*v*) were added to the 250 µL of a single collected fraction, then vortexed for 10 s and centrifuged at 11,000× *g*, 4 °C for 10 min. The aqueous phase was transferred to new tubes, where 350 μL of isopropanol and 100 μL of 0.8 M sodium acetate were added. The samples were thoroughly mixed and incubated at 4 °C for 30 min, with subsequent centrifugation at 11,000× *g*, 4 °C for 10 min. The supernatant was discarded. The pellet was washed with 1 mL of 70% ethanol, followed by centrifugation at 11,000× *g*, 4 °C for 10 min. In the end, the pellet was resuspended in 20 µL of DEPC H_2_O.

The extracted RNA samples were loaded onto an agarose gel and analyzed using an Agilent 2100 Bioanalyzer and RNA 6000 nano kit, according to the instructions of the manufacturer (Agilent Technologies, Santa Clara, CA, USA) and described previously [67,68]. The microfluidic UV-traces were single-sample scaled to assess rRNA composition of each fraction. This process does not support the comparison of rRNA abundances between fractions. Identification of rRNA species was according to Tiller and collaborators [36].

#### 3.3.2. Protein Purification and Concentration by Methanol/Chloroform Precipitation

A 2 mg/mL bovine serum albumin (BSA) stock solution was prepared from chromatographically purified BSA (Sigma Aldrich, Munich, Germany) as an internal reference standard for proteomic analyses. Then, 250 μL from a single fraction and 6 µL of BSA stock solution were mixed with 600 μL of methanol. After thorough mixing, 150 μL chloroform and 450 μL water were added. After subsequent vortex-mixing and immediate centrifugation for 20 min at 14,000 rpm and 4 °C, a white disc of protein formed between the lower organic layer and the upper aqueous layer. We discarded the upper aqueous layer, added 650 μL of methanol to the tube, and inverted the tubes three times. The tubes were centrifuged again for 20 min at identical settings. All liquid was carefully removed, and the pellet was air-dried. The precipitated dried protein was resuspended in SDS-PAGE sample buffer for electrophoretic analysis and can be submitted directly to proteomic analysis by liquid chromatography mass spectrometry (LCMS).

#### 3.3.3. Protein Purification and Concentration by Ultra-Membrane Centrifugation

We optimized this step for ribosome-associated proteome analysis to reduce the effects of varying amounts of residual sucrose in the final protein preparations. Here, 3 kDa cut-off Amicon Ultra-0.5 Ultracel-3 membrane centrifugal filters were used for protein purification and elution steps (Order number UFC500396; Merck KGaA, Darmstadt, Germany). Approximately 250 µL of a single sucrose fraction of interest was mixed with 6 µL of 2 mg/mL BSA internal standard. The mixture was then added to the filters and a volume of 500 µL adjusted by 1x salt and buffer solution for sucrose gradients. Centrifugation was for 20 min at 14,000 rpm at 4 °C. We repeated the buffer-washing step four times and adjusted the sample volume to 500 µL with sucrose free 1x salt and buffer solution. In a final step, 20 µL of the concentrated proteins was retrieved for proteomic analysis.

#### 3.3.4. Western Blot Analysis 

Western blot analysis was performed as described previously [69] using 12% (*w*/*v*) acrylamide SDS-PAGE. Polyclonal anti-RPL13B antibodies (AS13 2650/anti-L13-1), directed against the 23.8 kDa RPL13B/eL13 LSU protein encoded by At3g49010, and anti-RPS14 antibodies (AS12 2111/anti-RPS14-1) directed against the 16 kDa RPS14-1 (uS11) SSU protein encoded by AT2G36160), were obtained from Agrisera AB, Vännäs, Sweden [19,21]. The primary antibodies were diluted 1:2500 and detected by an anti-rabbit immunoglobulin G-horseradish peroxidase antibody that was diluted 1:10,000. Protein–antibody complexes were visualized by enhanced chemiluminescence (ECL) reagents (ThermoFisher Scientific Life Technologies GmbH, Darmstadt, Germany) and analyzed by the G:BOX F3 automated gel-imaging system (Syngene, Cambridge, UK). Images were processed with the Molecular Analyst™ software (Bio-Rad Laboratories Inc., Hercules, CA, USA).

#### 3.3.5. Proteome Analysis by Liquid Chromatography Mass Spectrometry Analysis

The following protocol entails protein digestion peptide clean-up and instrumental analysis. The disulfide bridges of proteins were reduced using a 200 mM DTT, 50 mM Tris-HCl pH 8.0 solution by incubation for 2 h at 25 °C. The thiol groups were alkylated using 200 mM iodoacetamide (IAM) by incubation for 1 h at 25 °C in the dark. The enzymatic digest was performed for 16 h at 30 °C using a Trypsin/endoproteinase LysC mix (Promega Corp., Fitchburg, WI) in a ratio of 25 parts of the protein to 1 part (*w*/*w*) of the protease. The digested peptides were acidified to pH < 3.0 with 10% trifluoroacetic acid (TFA). Prior to the chromatography, the peptide mixture was purified and desalted on C18 SEP-Pak columns (Waters, Milford, MA, USA), which were attached to a QIAvac 24 Plus (QIAGEN) vacuum manifold. The columns were equilibrated with 1 mL 100% methanol, once with 1 mL 80% acetonitrile (ACN) and twice with 1 mL of 0.1% TFA. The peptides were applied to the C18 SEP-Pak column and allowed to pass through slowly. The column was washed twice with 1 mL of 0.1% TFA. The peptides were eluted with 800 µL of a mixture of 60% ACN and 0.1% TFA, dried in a speed vacuum concentrator, and stored at −80 °C prior to mass spectrometry analysis. The peptides were resuspended in 30 μL of resuspension buffer, that is, 5% (*v*/*v*) acetonitrile and 2% (*v*/*v*) trifluoroacetic acid.

Measurements were performed by a Q Exactive HF Quadrupol-Orbitrap Mass Spectrometer (Thermo Fisher Scientific, Waltham, MA, USA) coupled to an ACQUITY UPLC M-Class System (Waters, Milford, MA, USA). Here, 8 μL samples were loaded onto an ACQUITY UPLC M-Class HSS T3 column, 75 μm inner diameter, 20 cm length, and 1.8 µm bead size (Waters, Milford, MA, USA) at a flow rate of 0.4 μL min^−1^ in a solution consisting of 3% (*v*/*v*) acetonitrile and 0.5% (*v*/*v*) formic acid. Peptide elution was facilitated by increasing the acetonitrile gradient from 3% to 24% (*v*/*v*) over 90 min, from 24% to 36% for the next 30 min, and from 36% to 85% for the last 6 min at a flow rate of 0.3 μL min^−1^. Peptide ions were detected in full scan mode, with range of mass-to-charge ratios from 300 to 1600 at a resolution of 120,000, automatic gain control (AGC) target of 3 × 10^6^, and maximum injection time IT of 100 ms. Each dd-MS2 scan was recorded in profile mode at a resolution of 15,000 with AGC target of 1 × 10^5^, isolation width mass-to-charge ratio 1.2 m/z and maximum IT of 150 ms.

Peptides for which MS/MS spectra had been recorded were excluded from further MS/MS scans for 30 s. Raw files were submitted to MaxQuant software for protein identification and quantification [70]. *Arabidopsis thaliana* TAIR10 protein sequences (35,386 entries) were used by the search engine Andromeda [71] for identification of peptides. The settings used for the search were as follows: 10 ppm peptide mass tolerance; 0.8 Da MS/MS tolerance; maximum of two missed cleavages allowed. The false discovery rate of both peptides and proteins was set to 0.01 using a decoy database. Carbamidomethylation of cysteine was set as a fixed modification and the minimum peptide length of seven amino acids was used. The “label-free quantification” (LFQ) option was selected for quantification. The quantification was performed of proteins with minimum of one unique and one razor peptide. Known contaminants, such as keratins, were removed from further analysis. Ribosomal proteins, biogenesis, and translation-related factors were annotated using the Majority.protein.ID read out of the MaxQuant analysis. Annotation of these proteins identifiers was supported by a list of gene identifiers, gene names, subunit memberships, and gene model descriptions compiled and collated from a set of reference publications that combined genome annotation with proteomic analyses of purified or enriched plant ribosome complexes [5,7,8,9,10,20,21,44,45,72,73,74,75].

The mass spectrometry proteomics data have been deposited to the ProteomeXchange Consortium via the PRIDE [76] partner repository with the dataset identifier PXD019329.

### 3.4. Relative Protein Quantification and Gene-Ontology Enrichment Analysis 

LFQ intensities of all proteins in a single fraction from the generated data sets were normalized to the abundance of the BSA standard in each fraction. For comparison between fractions, the relative abundance of each protein was calculated as the ratio of the normalized LFQ intensity in a single fraction to its average normalized LFQ intensity across all fractions of a data set. Log_2_-transformed ratios and corresponding gene identifiers were uploaded into the bioinformatics tool agriGo v2.0 to perform a gene ontology enrichment analysis [77,78].

## 4. Conclusions

Our analyses demonstrate that the established methodology allows separation of cytosolic and chloroplast non-translating ribosome complexes from respective monosomes and low oligomeric polysomes. The non-translating chloroplast and cytosolic 50S and 60S LSUs are separated, but most cytosolic and organelle ribo-complexes co-purify. The co-purification restricts relative quantification of ribosome complexes from leaf material using UV absorbance profiles of sucrose density fractionations [4,5,6,34]. Proteomic analysis is required for this purpose. However, UV absorbance profiles may serve as a proxy of relative quantification of cytosolic ribosome complexes in tissue, such as roots and non-germinating seeds that contain low relative amounts of organelle ribosomes [4,5,6,34]. Analysis of the relative abundance of RPs and ribosome complexes can be achieved from ~100 mg fresh weight. This amount supports the analysis of whole Arabidopsis root and shoot samples, but will require extensive pooling of material if applied to the analysis of leaf developmental stages or differentiated root zones.

In vivo formaldehyde crosslinking can be used for qualitative analysis of ribosome-associated proteins, as was exemplified by selected Arabidopsis pre-60S ribosome biogenesis factor homologs. However, in vivo cross-linked ribosome complexes clearly do not represent the in vivo composition of ribosome complexes and cannot serve as a proxy for analyzing the abundance and RP composition of non-translating ribosome complexes. In addition, the accumulation of translation initiation factors in the cross-linked 80S fraction raises the concern of crosslinking artifacts that may create non-native ribosome complexes.

Omitting crosslinking from the currently established combination of methods allows to assess the relative abundance of non-translating ribosome complexes and of RPs with high genomic coverage of cytosolic and organelle RP families and paralogs. Ribosome associated proteins that may be present and may change abundance in these complexes can be assessed by purification and concentration of sucrose density fractions using ultra-membrane centrifugation. Nevertheless, we cannot exclude that weakly bound RPs and ribosome associated proteins may still be lost or de-enriched from current preparations of ribosome complexes.

With this caveat in mind, indications of altered RP or RP paralog composition of non-translating plant ribosome complexes can be attempted. Proteome analyses of RP and RP paralog composition may support and extend our knowledge of altered ribo-proteomes. Such analyses may prove fruitful and important in view of recent studies that revealed unexpected and selective roles of core RPs from non-plant eukaryotic ribosomes in cell homeostasis and organism development [23]. We argue that direct evidence of plant ribosome heterogeneity at the level of core RPs is needed. Ribosome heterogeneity and unique functions of RPs or RP paralogs may shape ribosome populations and translation during organismal development and acclimation to stress [22,79].

## Figures and Tables

**Table 1:** Overview of annotated and detected ribosomal proteins (RPs) and protein families of *Arabidopsis thaliana*. The set of expected cytoplasmic and chloroplast RP families [7,10] and RP paralogs was combined from genome annotations and previous experimental data [20,21]. Listed mitochondrial RPs were confirmed by two independent studies [44,45]. LSU, large subunit; SSU, small subunit.

		Eukaryotic			Chloroplast			Mitochondrial		
	Total # of RPs	60S	40S	Total	50S	30S	Total	mtLSU	mtSSU	Total
**Expected RPs**	414	142	100	242	42	34	76	53	43	96
**Identified RPs**	216	81	63	144	33	25	58	14	-	14
**Expected RP families**	223	45	33	78	33	25	58	45	42	87
**Identified RP families**	142	41	33	74	30	25	55	13	-	13

**Table 2:** A detailed list of identified RPs containing gene identifier, subunit origin, and ribosomal protein nomenclature note. New and old RPs’ nomenclature were matched according to Ban et al. 2014 [7]. Presence of plastid proteins in chloroplast ribosomes previously determined by structure elucidation of the spinach 70S ribosome [10,51].

Gene Code	Ribosome Origin	Subunit Name	New RP Names (Ban et al. 2014) [7]	Old RP Names	Sormani et al., 2011 [21]	Hummel et al., 2015 [20]	Waltz et al., 2019 [44] and Rugen et al., 2019 [45]
AT1G08360	cytosolic	60S	uL1	RPL10aA	+	+	NA
AT2G27530	cytosolic	60S	uL1	RPL10aB	+	+	NA
AT5G22440	cytosolic	60S	uL1	RPL10aC	+	+	NA
AT2G18020	cytosolic	60S	uL2	RPL8A	+	+	NA
AT4G36130	cytosolic	60S	uL2	RPL8C	+	+	NA
AT1G43170	cytosolic	60S	uL3	RPL3A	+	+	NA
AT1G61580	cytosolic	60S	uL3	RPL3B	+	+	NA
AT3G09630	cytosolic	60S	uL4	RPL4A	+	+	NA
AT5G02870	cytosolic	60S	uL4	RPL4D	+	+	NA
AT5G45775	cytosolic	60S	uL5	RPL11D	+	+	NA
AT1G33140	cytosolic	60S	uL6	RPL9C	+	+	NA
AT4G10450	cytosolic	60S	uL6	RPL9D	+	+	NA
AT3G09200	cytosolic	60S	uL10	RPP0B	+	+	NA
AT2G37190	cytosolic	60S	uL11	RPL12A	+	+	NA
AT3G53430	cytosolic	60S	uL11	RPL12B	+	+	NA
AT5G60670	cytosolic	60S	uL11	RPL12C	+	+	NA
AT3G07110	cytosolic	60S	uL13	RPL13aA	+	+	NA
AT3G24830	cytosolic	60S	uL13	RPL13aB	+	+	NA
AT4G13170	cytosolic	60S	uL13	RPL13aC	+	+	NA
AT5G48760	cytosolic	60S	uL13	RPL13aD	+	+	NA
AT3G04400	cytosolic	60S	uL14	RPL23C	+	+	NA
AT1G70600	cytosolic	60S	uL15	RPL27aC	+	+	NA
AT1G14320	cytosolic	60S	uL16	RPL10A	+	+	NA
AT1G66580	cytosolic	60S	uL16	RPL10C	+	+	NA
AT3G25520	cytosolic	60S	uL18	RPL5A	+	+	NA
AT5G39740	cytosolic	60S	uL18	RPL5B	+	+	NA
AT1G27400	cytosolic	60S	uL22	RPL17A	+	+	NA
AT1G67430	cytosolic	60S	uL22	RPL17B	+	+	NA
AT3G55280	cytosolic	60S	uL23	RPL23aB	+	+	NA
AT3G49910	cytosolic	60S	uL24	RPL26A	+	+	NA
AT5G67510	cytosolic	60S	uL24	RPL26B	+	+	NA
AT3G09500	cytosolic	60S	uL29	RPL35A	+	+	NA
AT5G02610	cytosolic	60S	uL29	RPL35D	+	+	NA
AT2G01250	cytosolic	60S	uL30	RPL7B	+	+	NA
AT2G44120	cytosolic	60S	uL30	RPL7C	+	+	NA
AT3G13580	cytosolic	60S	uL30	RPL7D	+	+	NA
AT1G18540	cytosolic	60S	eL6	RPL6A	+	+	NA
AT1G74050	cytosolic	60S	eL6	RPL6C	+	+	NA
AT3G62870	cytosolic	60S	eL8	RPL7aB	+	+	NA
AT3G49010	cytosolic	60S	eL13	RPL13B	+	+	NA
AT5G23900	cytosolic	60S	eL13	RPL13D	+	+	NA
AT2G20450	cytosolic	60S	eL14	RPL14A	+	+	NA
AT4G27090	cytosolic	60S	eL14	RPL14B	+	+	NA
AT4G16720	cytosolic	60S	eL15	RPL15A	+	+	NA
AT3G05590	cytosolic	60S	eL18	RPL18B	+	+	NA
AT5G27850	cytosolic	60S	eL18	RPL18C	+	+	NA
AT1G02780	cytosolic	60S	eL19	RPL19A	+	+	NA
AT3G16780	cytosolic	60S	eL19	RPL19B	+	+	NA
AT4G02230	cytosolic	60S	eL19	RPL19C	+	+	NA
AT2G34480	cytosolic	60S	eL20	RPL18aB	+	+	NA
AT3G14600	cytosolic	60S	eL20	RPL18aC	+	+	NA
AT1G57860	cytosolic	60S	eL21	RPL21G	+	+	NA
AT3G05560	cytosolic	60S	eL22	RPL22B	+	+	NA
AT5G27770	cytosolic	60S	eL22	RPL22C	+	+	NA
AT3G53020	cytosolic	60S	eL24	RPL24B	+	+	NA
AT3G22230	cytosolic	60S	eL27	RPL27B	+	+	NA
AT4G15000	cytosolic	60S	eL27	RPL27C	+	+	NA
AT2G19730	cytosolic	60S	eL28	RPL28A	+	+	NA
AT4G29410	cytosolic	60S	eL28	RPL28C	+	+	NA
AT3G18740	cytosolic	60S	eL30	RPL30C	+	+	NA
AT5G56710	cytosolic	60S	eL31	RPL31C	+	+	NA
AT4G18100	cytosolic	60S	eL32	RPL32A	+	+	NA
AT5G46430	cytosolic	60S	eL32	RPL32B	+	+	NA
AT1G41880	cytosolic	60S	eL33	RPL35aB	+	+	NA
AT3G55750	cytosolic	60S	eL33	RPL35aD	+	+	NA
AT1G69620	cytosolic	60S	eL34	RPL34B	+	+	NA
AT3G28900	cytosolic	60S	eL34	RPL34C	+	+	NA
AT2G37600	cytosolic	60S	eL36	RPL36A	+	+	NA
AT5G02450	cytosolic	60S	eL36	RPL36C	+	+	NA
AT3G59540	cytosolic	60S	eL38	RPL38B	+	+	NA
AT4G14320	cytosolic	60S	eL42	RPL36aB	+	+	NA
AT3G60245	cytosolic	60S	eL43	RPL37aC	+	+	NA
AT1G01100	cytosolic	60S	P1/P2	RPP1A	+	+	NA
AT4G00810	cytosolic	60S	P1/P2	RPP1B	+	+	NA
AT5G47700	cytosolic	60S	P1/P2	RPP1C	+	+	NA
AT2G27720	cytosolic	60S	P1/P2	RPP2A	+	+	NA
AT2G27710	cytosolic	60S	P1/P2	RPP2B	+	+	NA
AT3G28500	cytosolic	60S	P1/P2	RPP2C	+	+	NA
AT3G44590	cytosolic	60S	P1/P2	RPP2D	+	+	NA
AT4G25890	cytosolic	60S	P1/P2	RPP3A	+	+	NA
AT5G57290	cytosolic	60S	P1/P2	RPP3B	+	+	NA
AT1G72370	cytosolic	40S	uS2	RPSaA	+	+	NA
AT2G31610	cytosolic	40S	uS3	RPS3A	+	+	NA
AT3G53870	cytosolic	40S	uS3	RPS3B	+	+	NA
AT5G35530	cytosolic	40S	uS3	RPS3C	+	+	NA
AT5G15200	cytosolic	40S	uS4	RPS9B	+	+	NA
AT1G59359	cytosolic	40S	uS5	RPS2B	+	+	NA
AT2G41840	cytosolic	40S	uS5	RPS2C	+	+	NA
AT2G37270	cytosolic	40S	uS7	RPS5A	+	+	NA
AT3G11940	cytosolic	40S	uS7	RPS5B	+	+	NA
AT5G59850	cytosolic	40S	uS8	RPS15aF	+	+	NA
AT2G09990	cytosolic	40S	uS9	RPS16A	+	+	NA
AT5G18380	cytosolic	40S	uS9	RPS16C	+	+	NA
AT3G47370	cytosolic	40S	uS10	RPS20B	+	+	NA
AT5G62300	cytosolic	40S	uS10	RPS20C	+	+	NA
AT2G36160	cytosolic	40S	uS11	RPS14A	+	+	NA
AT3G11510	cytosolic	40S	uS11	RPS14B	+	+	NA
AT3G52580	cytosolic	40S	uS11	RPS14C	+	+	NA
AT5G02960	cytosolic	40S	uS12	RPS23B	+	+	NA
AT4G09800	cytosolic	40S	uS13	RPS18C	+	+	NA
AT4G33865	cytosolic	40S	uS14	RPS29C	+	+	NA
AT4G00100	cytosolic	40S	uS15	RPS13B	+	+	NA
AT3G48930	cytosolic	40S	uS17	RPS11A	+	+	NA
AT5G23740	cytosolic	40S	uS17	RPS11C	+	+	NA
AT1G04270	cytosolic	40S	uS19	RPS15A	+	+	NA
AT5G09500	cytosolic	40S	uS19	RPS15C	+	+	NA
AT5G09510	cytosolic	40S	uS19	RPS15D	+	+	NA
AT3G04840	cytosolic	40S	eS1	RPS3aA	+	+	NA
AT4G34670	cytosolic	40S	eS1	RPS3aB	+	+	NA
AT5G07090	cytosolic	40S	eS4	RPS4B	+	+	NA
AT5G58420	cytosolic	40S	eS4	RPS4D	+	+	NA
AT4G31700	cytosolic	40S	eS6	RPS6A	+	+	NA
AT5G10360	cytosolic	40S	eS6	RPS6B	+	+	NA
AT1G48830	cytosolic	40S	eS7	RPS7A	+	+	NA
AT3G02560	cytosolic	40S	eS7	RPS7B	+	+	NA
AT5G16130	cytosolic	40S	eS7	RPS7C	+	+	NA
AT5G20290	cytosolic	40S	eS8	RPS8A	+	+	NA
AT4G25740	cytosolic	40S	eS10	RPS10A	+	+	NA
AT5G41520	cytosolic	40S	eS10	RPS10B	+	+	NA
AT5G52650	cytosolic	40S	eS10	RPS10C	+	+	NA
AT1G15930	cytosolic	40S	eS12	RPS12A	+	+	NA
AT2G32060	cytosolic	40S	eS12	RPS12C	+	+	NA
AT2G05220	cytosolic	40S	eS17	RPS17B	+	+	NA
AT5G04800	cytosolic	40S	eS17	RPS17D	+	+	NA
AT3G02080	cytosolic	40S	eS19	RPS19A	+	+	NA
AT5G61170	cytosolic	40S	eS19	RPS19C	+	+	NA
AT3G53890	cytosolic	40S	eS21	RPS21B	+	+	NA
AT5G27700	cytosolic	40S	eS21	RPS21C	+	+	NA
AT3G04920	cytosolic	40S	eS24	RPS24A	+	+	NA
AT5G28060	cytosolic	40S	eS24	RPS24B	+	+	NA
AT2G21580	cytosolic	40S	eS25	RPS25B	+	+	NA
AT4G39200	cytosolic	40S	eS25	RPS25E	+	+	NA
AT2G40510	cytosolic	40S	eS26	RPS26A	+	+	NA
AT2G40590	cytosolic	40S	eS26	RPS26B	+	+	NA
AT3G56340	cytosolic	40S	eS26	RPS26C	+	+	NA
AT2G45710	cytosolic	40S	eS27	RPS27A	+	+	NA
AT5G47930	cytosolic	40S	eS27	RPS27D	+	+	NA
AT5G03850	cytosolic	40S	eS28	RPS28B	+	+	NA
AT5G64140	cytosolic	40S	eS28	RPS28C	+	+	NA
AT5G56670	cytosolic	40S	eS30	RPS30C	+	+	NA
AT1G23410	cytosolic	40S	eS31	RPS27aA	+	+	NA
AT1G18080	cytosolic	40S	RACK1	RACK1A	−	+	NA
AT1G48630	cytosolic	40S	RACK1	RACK1B	−	+	NA
AT3G18130	cytosolic	40S	RACK1	RACK1C	−	+	NA
AT3G63490	plastid	50S	uL1c	RPL1	+	−	NA
ATCG01310	plastid	50S	uL2c	RPL2.2	+	−	NA
AT2G43030	plastid	50S	uL3c	RPL3 related	+	−	NA
AT1G07320	plastid	50S	uL4c	RPL4	+	−	NA
AT4G01310	plastid	50S	uL5c	RPL5 family	+	−	NA
AT1G05190	plastid	50S	uL6c	RPL6/emb2394	+	−	NA
AT3G44890	plastid	50S	bL9c	RPL9/CL9	+	−	NA
AT5G13510	plastid	50S	uL10c	RPL10	+	−	NA
AT1G32990	plastid	50S	uL11c	RPL11	+	−	NA
AT3G27850	plastid	50S	bL12c	RPL12C	+	−	NA
AT1G78630	plastid	50S	uL13c	RPL13A	+	−	NA
ATCG00780	plastid	50S	uL14c	RPL14/HLL	+	−	NA
AT3G25920	plastid	50S	uL15c	RPL15	+	−	NA
ATCG00790	plastid	50S	uL16c	RPL16	+	−	NA
AT3G54210	plastid	50S	bL17c	RPL17	+	−	NA
AT3G20230	plastid	50S	uL18c	RPL18N	+	−	NA
AT5G13720	plastid	50S	uL18c	RPL18N	+	−	NA
AT1G48350	plastid	50S	uL18c	RPL18N	+	−	NA
AT4G17560	plastid	50S	bL19c	RPL19	+	−	NA
AT5G47190	plastid	50S	bL19c	RPL19	+	−	NA
ATCG00660	plastid	50S	bL20c	RPL20	+	−	NA
AT1G35680	plastid	50S	bL21c	RPL21/CL21	+	−	NA
ATCG00810	plastid	50S	uL22c	RPL22	+	−	NA
ATCG01300	plastid	50S	uL23c	RPL23.2	+	−	NA
AT5G54600	plastid	50S	uL24c	RPL24	+	−	NA
AT5G40950	plastid	50S	bL27c	RPL27	+	−	NA
AT2G33450	plastid	50S	bL28c	RPL28	+	−	NA
AT5G65220	plastid	50S	uL29c	RPL29	+	−	NA
AT1G75350	plastid	50S	bL31c	RPL31	+	−	NA
ATCG01020	plastid	50S	bL32c	RPL32	+	−	NA
AT2G24090	plastid	50S	bL35c	RPL35 family	+	−	NA
AT3G56910	plastid	50S	cL37	PSRP-5	+	−	NA
AT5G17870	plastid	50S	cL38	PSRP-6	+	−	NA
AT5G30510	plastid	30S	bS1c	RPS1	+	−	NA
ATCG00160	plastid	30S	uS2c	RPS2	+	−	NA
ATCG00800	plastid	30S	uS3c	RPS3aN	+	−	NA
ATCG00380	plastid	30S	uS4c	RPS4	+	−	NA
AT2G33800	plastid	30S	uS5c	RPS5	+	−	NA
AT1G64510	plastid	30S	bS6c	RPS6	+	−	NA
ATCG01240	plastid	30S	uS7c	RPS7.1	+	−	NA
ATCG00770	plastid	30S	uS8c	RPS8	+	−	NA
AT1G74970	plastid	30S	uS9c	RPS9	+	−	NA
AT3G13120	plastid	30S	uS10c	RPS10	+	−	NA
ATCG00750	plastid	30S	uS11c	RPS11	+	−	NA
ATCG01230	plastid	30S	uS12c	RPS12	+	−	NA
AT5G14320	plastid	30S	uS13c	RPS13	+	−	NA
ATCG00330	plastid	30S	uS14c	RPS14	+	−	NA
ATCG01120	plastid	30S	uS15c	RPS15	+	−	NA
AT4G34620	plastid	30S	bS16c	RPS16	+	−	NA
AT1G79850	plastid	30S	uS17c	RPS17	+	−	NA
ATCG00650	plastid	30S	bS18c	RPS18	+	−	NA
ATCG00820	plastid	30S	uS19c	RPS19	+	−	NA
AT3G15190	plastid	30S	bS20c	RPS20	+	−	NA
AT3G27160	plastid	30S	bS21c	RPS21 GHS1	+	−	NA
AT2G38140	plastid	30S	bTHXc	PSRP-4	+	−	NA
AT3G52150	plastid	30S	cS22	PSRP-2	+	−	NA
AT1G68590	plastid	30S	cS23	PSRP-3	+	−	NA
AT5G24490	plastid	30S	plastid pY	PSRP-1	+	−	NA
AT4G29060	RP homolog			PSRP-7	+	−	NA
AT2G42710	Mitochondrial	mtLSU	uL1m	RPL1	+	−	+
AT2G44065	Mitochondrial	mtLSU	uL2m	RPL2	+	−	+
AT2G20060	Mitochondrial	mtLSU	uL4m	RPL4	+	−	+
AT3G01790	Mitochondrial	mtLSU	uL13m	RPL13	+	−	+
AT5G46160	Mitochondrial	mtLSU	uL14m	RPL14/HLP	+	−	+
AT5G64670	Mitochondrial	mtLSU	uL15m	RPL15	+	−	+
AT5G53070	Mitochondrial	mtLSU	bL9m	RPL9	+	−	+
AT4G30930	Mitochondrial	mtLSU	bL21m	RPL21/NFD1	+	−	+
AT4G23620	Mitochondrial	mtLSU	bL25m	RPL15	+	−	+
AT5G66860	Mitochondrial	mtLSU	bL25m	RPL25	+	−	+
AT4G05400	Mitochondrial	mtLSU	mL40	Cu binding	−	−	+
AT1G60770	Mitochondrial	mtLSU	mL101(rPPR4)	PPR	−	−	+
AT5G60960	Mitochondrial	mtLSU	mL104(rPPR9)	PPR (PNM1)	−	−	+
AT1G73940	Mitochondrial	mtLSU	mL106	TNF protein	−	−	+

+ Described by the authors; − Not described by the authors; NA—non-applicable.

**Table 3:** Preparation of ribosome extraction buffer (REB). DTT, dithiothreitol; PMSF, phenylmethylsulfonyl fluoride; DEPC, diethyl pyrocarbonate; PTE, polyoxyethylene 10 tridecyl ether; DOC, sodium deoxycholate.

Ribosome Extraction Buffer	Chemical Components (Final Concentration)	Stock Solutions (Concentration)	Volume Required for 5 mL	Volume Required for 50 mL	Temperature Conditions
**Pre-REB** Prepare 5.0 mL aliquots and store less than 6 months at −20 °C.	200 mM Tris-HCl	2.0 M (pH 9.0)	-	5.0 mL	rt
	200 mM KCl	2.0 M	-	5.0 mL	rt
	25 mM EGTA	0.5 M (pH 8.3)	-	2.5 mL	rt
	36 mM MgCl_2_	1.0 M	-	1.8 mL	rt
	DEPC–H_2_O	-	-	adjust to 50 mL	rt
	1% Detergent mix	20% (*v*/*v* or *w*/*v*)	-	2.5 mL	45 °C
	1% (*v*/*v*) PTE	20% (*v*/*v*)	-	2.5 mL	rt
	1% (*w*/*v*) DOC	10% (*w*/*v*)	-	5.0 mL	rt
**Final REB** Prepare freshly at day of use	50 µg/mL Cycloheximide	50 mg/mL	5 µL	-	4 °C
	50 µg/mL Chloramphenicol	50 mg/mL	5 µL	-	4 °C
	1 mg/mL Heparin	200 mg/mL	25 µL	-	4 °C
	5 mM DTT	1 M	25 µL	-	4 °C
	1 mM PMSF	0.5 M	10 µL	-	4 °C
	complete protease inhibitor cocktail	1 tablet/mL	10 µL	-	4 °C

**Table 4:** Preparation of 15% to 60% (*w*/*v*) sucrose gradients in single large or small ultracentrifugation tubes.

Number of Tubes (n)	Sucrose Gradient Solution (%)	2 M Sucrose (mL)	10 × Salt and Buffer Solution (mL)	ddH_2_O (mL)	Chloramphenicol (μL)	Cycloheximide (μL)	Final Volume (mL)	Pipetting Order	Large Tubes (mL)	Small Tubes (mL)
**1**	60	1.31	0.15	0.04	0.15	0.15	1.8	1	1.5	0.75
	45	1.97	0.30	0.73	0.30	0.30	3.6	2	3.0	1.50
	30	1.31	0.30	1.39	0.30	0.30	3.6	3	3.0	1.50
	15	0.33	0.15	1.02	0.15	0.15	1.8	4	1.5	0.75

## Supplementary Materials

The following are available online at https://www.mdpi.com/2223-7747/9/7/892/s1, Figure S1. Hydroponic cultivation system for the growth and harvest of axenic Arabidopsis thaliana root and shoot materials; Figure S2. Ribosome sedimentation profile of Arabidopsis thaliana rosette leaf tissue corresponding to the proteomic analyses of Figure 3; Figure S3. Profile of detected mitochondrial RPs from leaf material plotted as sum of LFQ intensities; Figure S4. High-resolution version of the heat map shown in Figure 5D; Table S1. List of ribosomal proteins that were correlated to a choice of subunit reference proteins.
