# Supplementary material for: Separation and Paired Proteome Profiling of Plant Chloroplast and Cytoplasmic Ribosomes
Source: Plants (Basel). 2020 Jul 14;9(7):892. doi: 10.3390/plants9070892 (PMC7411607; doi:10.3390/plants9070892)
Supplement: Supplementary file 1 [file plants-09-00892-s001.zip › plants-813283-supplementary/plants-813283-supplementary Figures.docx]

**Supplementary Figures:**

**
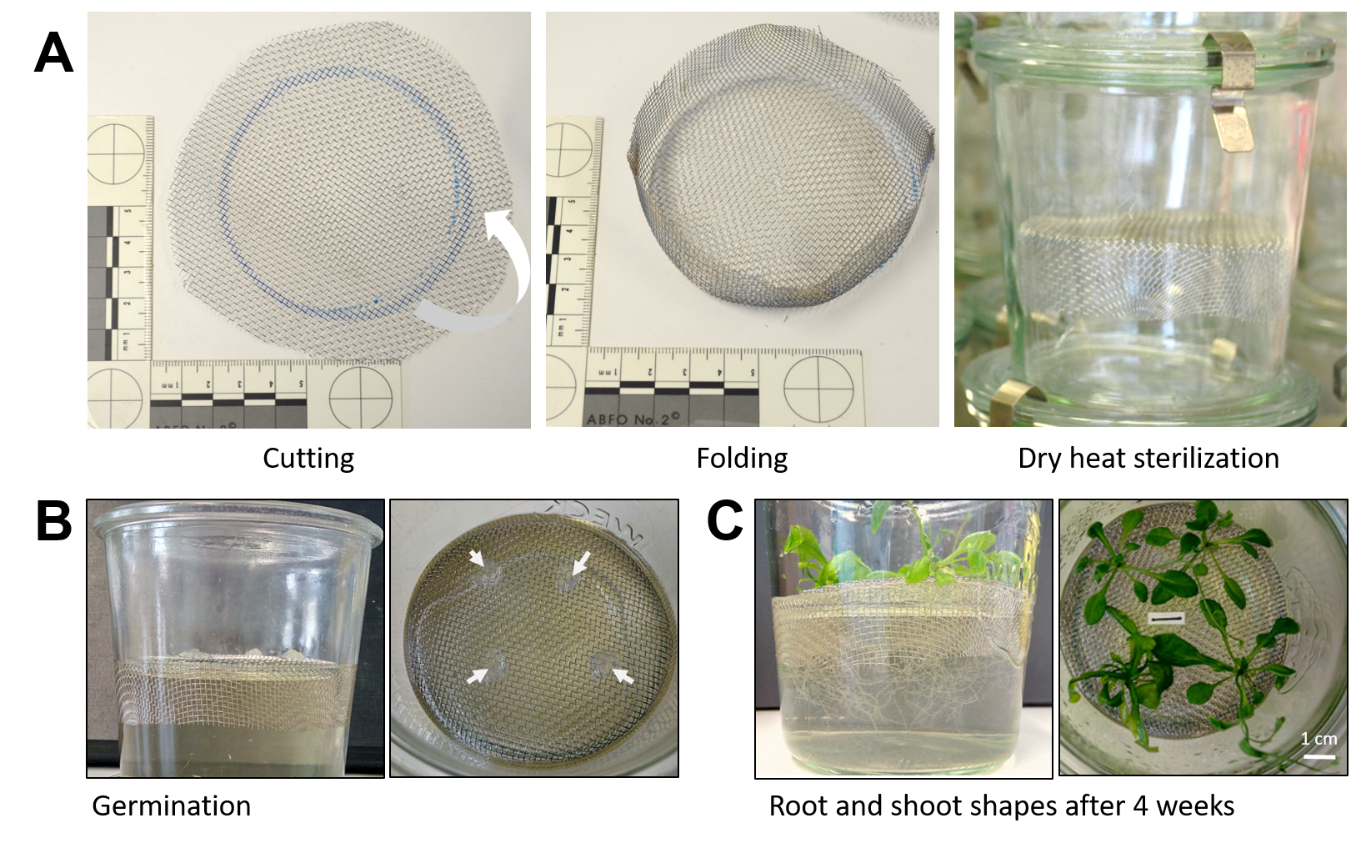
**

**Figure S1.** Hydroponic cultivation system for the growth and harvest of axenic *Arabidopsis thaliana* root and shoot materials. (**A**) “Trampoline” mesh preparation. Equal circular pieces of stainless-steel mesh (0.25 mm wire diameter and 1.4 mm mesh width) of ~11 cm diameter, that is, approximately the top diameter of a slightly tapered cultivation glass pot were prepared. The “trampoline” was folded using the bottom of a cultivation pot with ~8 cm diameter. This process created an ~1.5 cm overhang that was bent further inside. The mesh was fixed by tension at the mid-level of the cultivation pot. The assembled “trampoline” system was sterilized by dry heat autoclaving. (**B**) Seed placement and exemplary cultivation results. A volume of ~250 mL sterilized liquid Murashige and Skoog (MS) medium containing 2% (w/v) sucrose was filled into the glass pot and the trampoline adjusted to the liquid surface avoiding air bubbles below the mesh and submergence. Sterilized plant seeds were placed carefully onto the mesh using single small pieces of solid 2% sucrose MS medium per plant (arrows). The bottoms of cultivation pots up to liquid-level were covered, but root systems were not completely darkened. (**C**) Plant cultivation under long day conditions with 16 h/8 h (day/ night) and at 20 °C/18 °C (day/ night) generated plants at developmental stage ~1.10 [32] within 4 weeks.

**
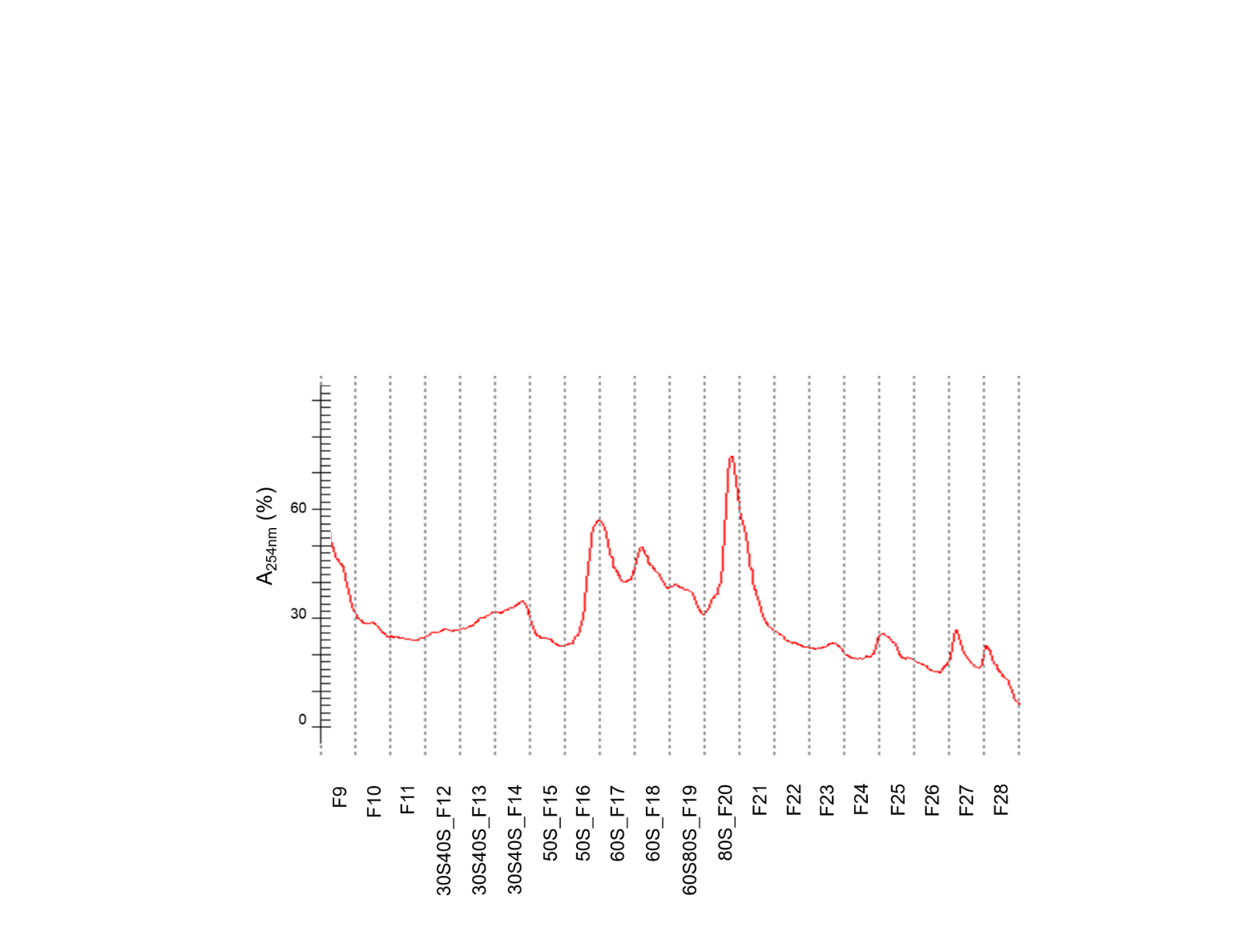
**

**Figure S2.** Ribosome sedimentation profile of *Arabidopsis thaliana* rosette leaf tissue corresponding to the proteomic analyses of Figure 3.


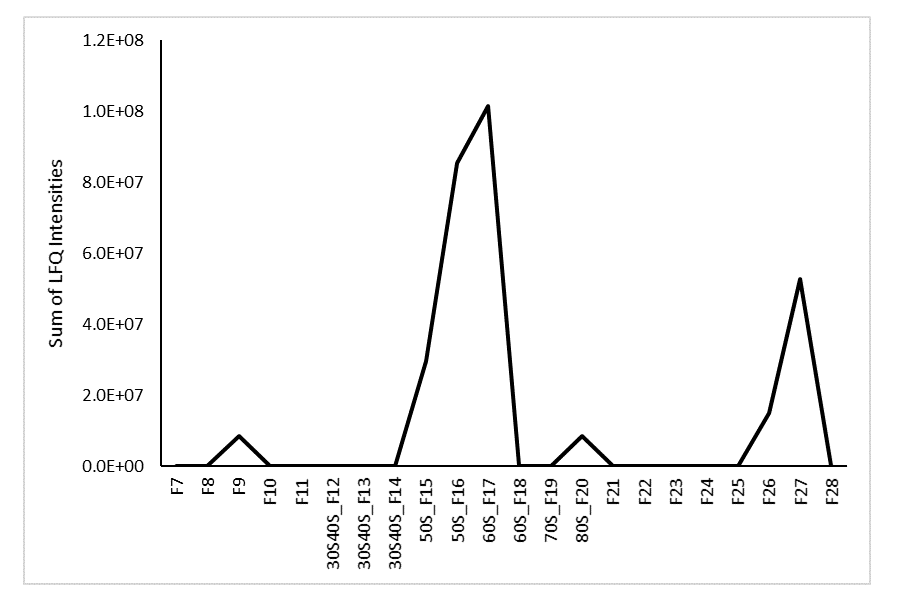


**Figure S3.** Profile of detected mitochondrial RPs from leaf material plotted as sum of LFQ intensities.


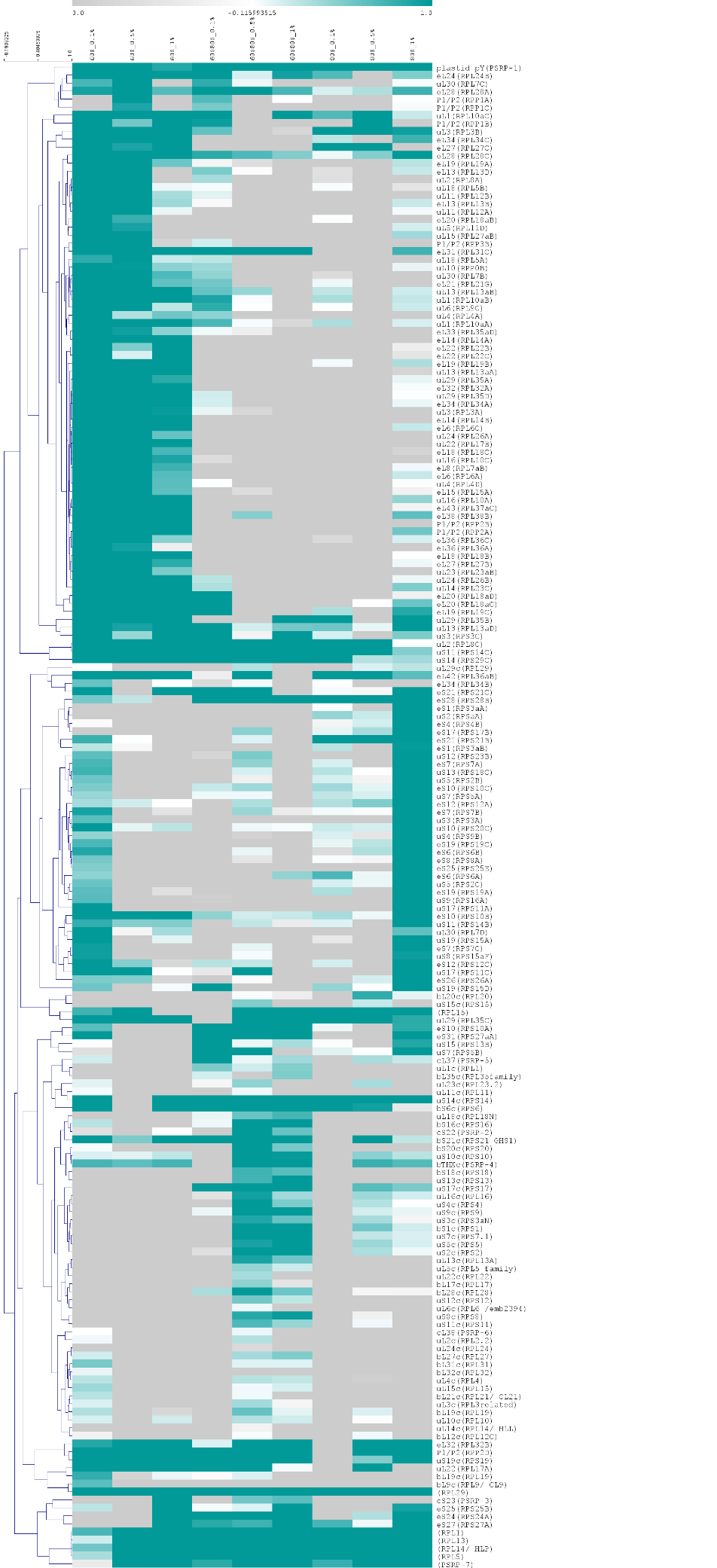


**Figure S4.** High-resolution version of the heat map shown in Figure 5D.
