# Supplementary figures and images for: Separation and Paired Proteome Profiling of Plant Chloroplast and Cytoplasmic Ribosomes
Source: Plants (Basel). 2020 Jul 14;9(7):892. doi: 10.3390/plants9070892 (PMC7411607; doi:10.3390/plants9070892)

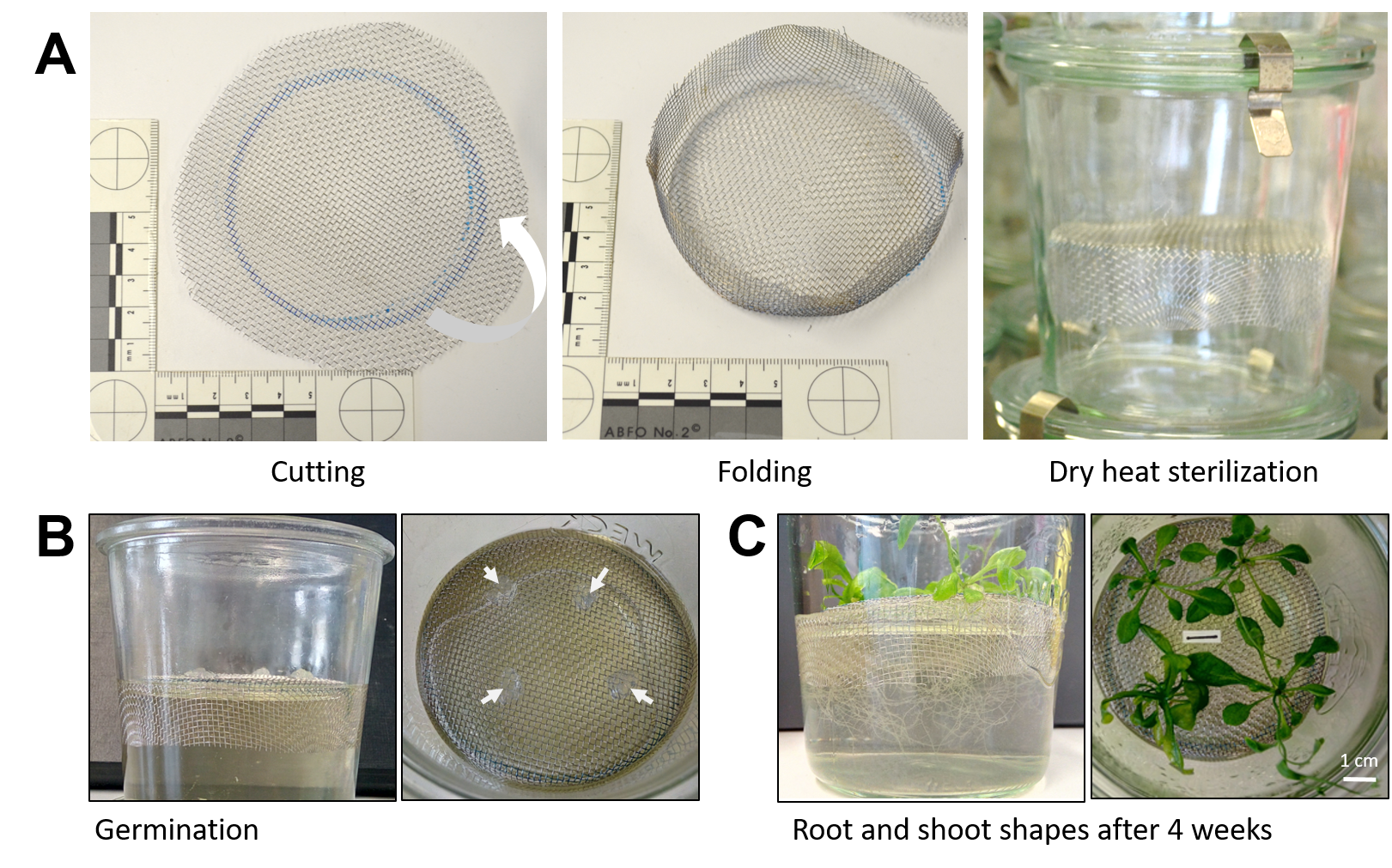

Supplement: Supplementary file 1 [file plants-09-00892-s001.zip › plants-813283-supplementary/Supplementary_Figure_S1.tif]

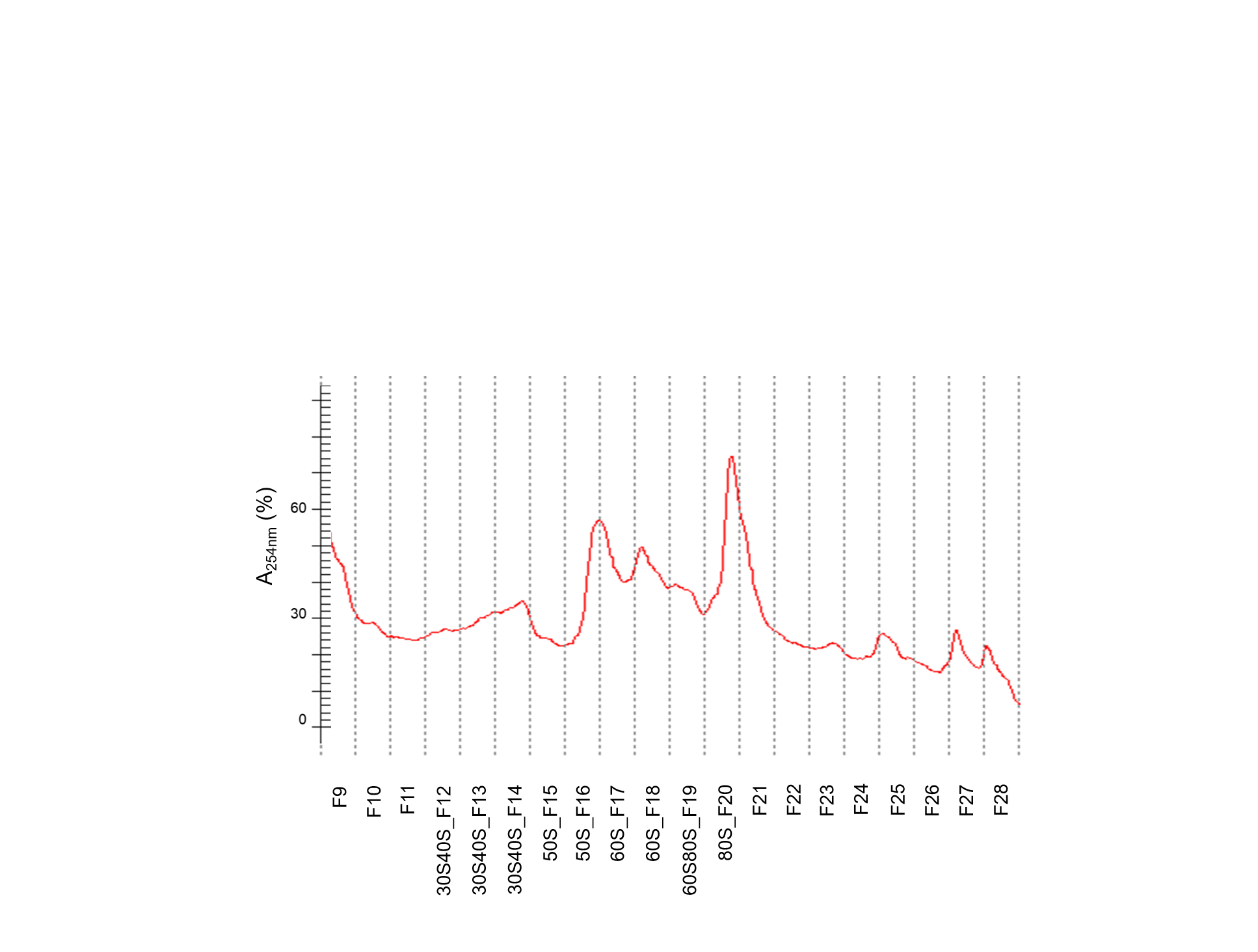

Supplement: Supplementary file 1 [file plants-09-00892-s001.zip › plants-813283-supplementary/Supplementary_Figure_S2.tif]

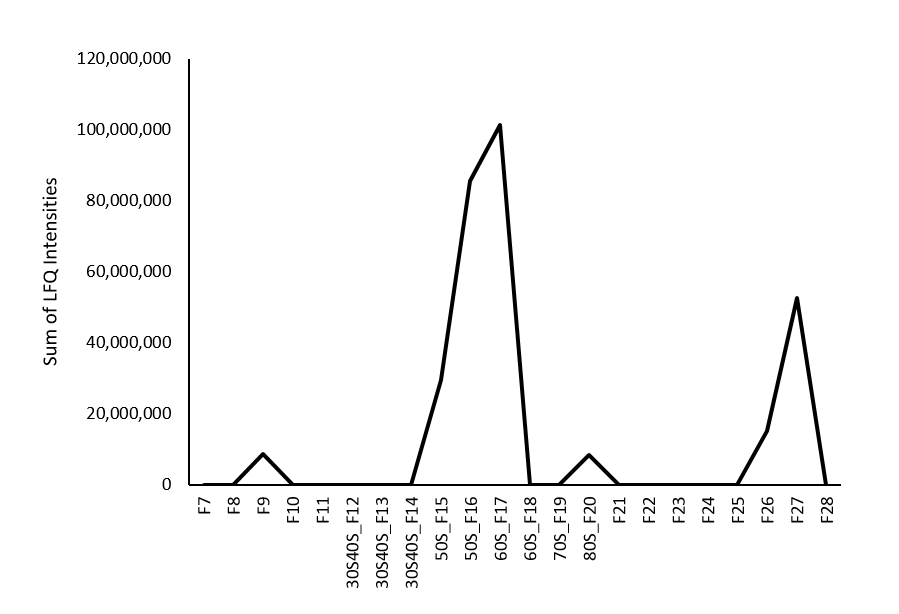

Supplement: Supplementary file 1 [file plants-09-00892-s001.zip › plants-813283-supplementary/Supplementary_Figure_S3.tif]

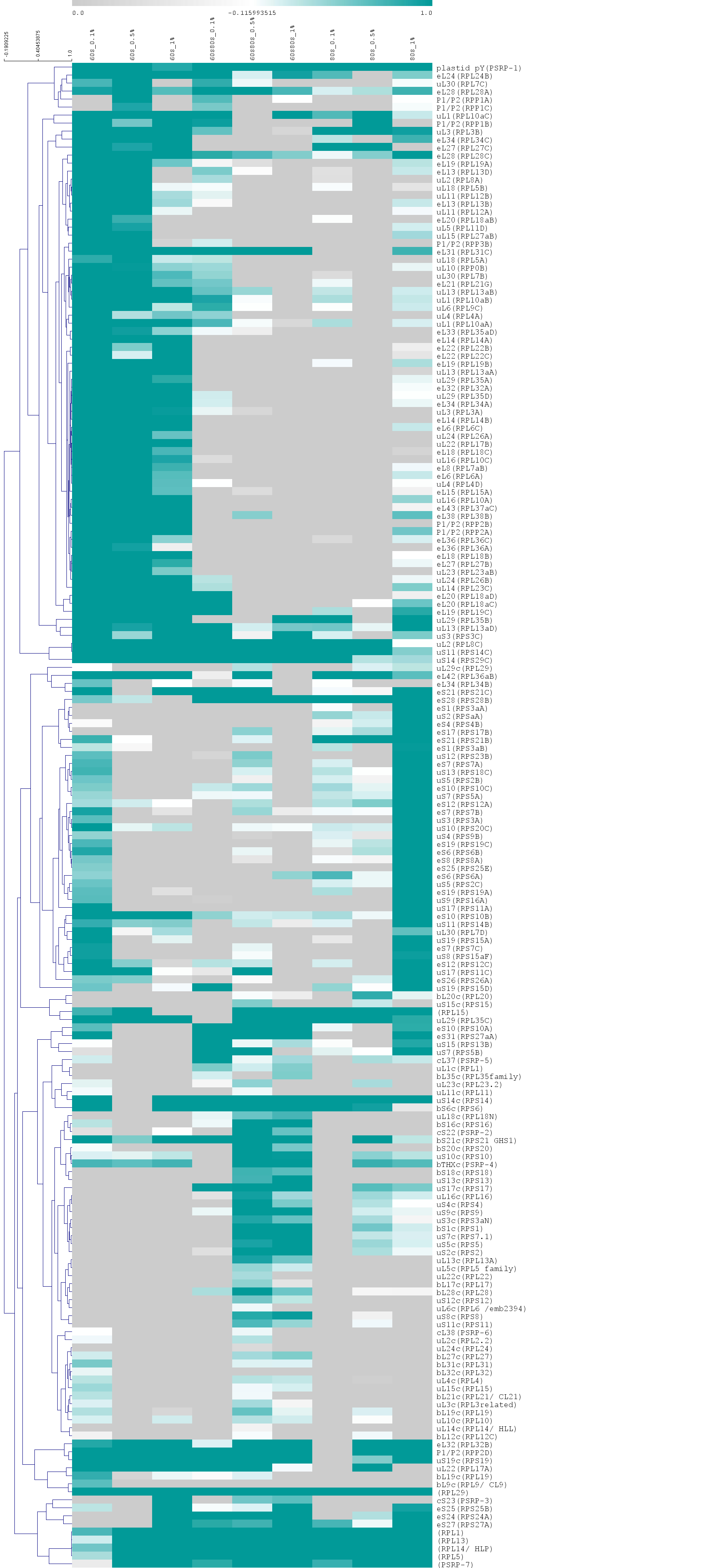

Supplement: Supplementary file 1 [file plants-09-00892-s001.zip › plants-813283-supplementary/Supplementary_Figure_S4.tif]
